# High morphological disparity in a bizarre Paleocene fauna of predatory freshwater reptiles

**DOI:** 10.1186/s12862-022-01985-z

**Published:** 2022-03-21

**Authors:** Chase Doran Brownstein

**Affiliations:** 1 Stamford Museum and Nature Center, Stamford, CT USA; 2 Department of Ecology and Evolutionary Biology, Yale University, New Haven, CT USA

**Keywords:** Choristodera, Phylogenetics, Cenozoic, K-Pg, Biogeography, Ecosystem recovery

## Abstract

**Background:**

The consequences of the K-Pg mass extinction are reflected across present biodiversity, but many faunas that appeared immediately after the extinction event were very different from current ones. Choristodera is a clade of reptiles of uncertain phylogenetic placement that have an extremely poor fossil record throughout their 150-million-year history. Yet, choristoderes survived the K-Pg event and persisted until the Miocene.

**Results:**

I describe the skulls and skeletons of two new choristoderes from a single Paleocene ecosystem in western North America that reveal the hidden Cenozoic diversity of this reptile clade. Despite their similar size, the new species deviate dramatically in morphology. *Kosmodraco magnicornis* gen. et sp. nov. possesses an extremely short snout and extensive cranial ornamentation. The sacrum of *K. magnicornis* bears enlarged muscle attachment sites and other modifications reminiscent of some giant crocodylians. In contrast, *Champsosaurus norelli* sp. nov. is a longirostrine species with an uninflated and ventrally divergent postorbital skull. Together with a North American choristodere previously classified in the European genus *Simoedosaurus*, *K. magnicornis* substantiates a new clade of giant, short-snouted taxa endemic to the Americas. *C. norelli* is found to be an early-diverging member of the genus *Champsosaurus* from the Cretaceous-Paleogene of the northern hemisphere. This suggests the presence of several ghost lineages of champsosaurid that crossed the K-Pg boundary.

**Conclusions:**

The new taxa greatly increase Cenozoic choristodere richness and strengthen the evidence for the existence of distinctive freshwater faunas in Paleogene Eurasia and North America, where this clade diversified to exploit newly available macropredatory niches in the aftermath of the asteroid impact. The new choristoderes also reveal the distinct ecological context in which extant freshwater predators of the Americas like alligatoroids and gars have their origins: Paleocene fluviolacustrine ecosystems in North America displayed high large predator diversity and morphological disparity relative to modern ones.

**Supplementary Information:**

The online version contains supplementary material available at 10.1186/s12862-022-01985-z.

## Introduction

With the recognition of several sites that document the stepwise changes different environments experienced over an order of years to tens of thousands of years following the Cretaceous-Paleogene mass extinction (e.g., [54]), how global ecosystems recovered following this event has seen reinvigorated attention. One important observation has been the appearance of biodiversity ‘oddities’ in early Paleocene ecosystems. These include giant snakes [46], large-bodied herbivorous galloanseran birds (e.g., [81]), and an unexpected diversity of archaic mammal groups (e.g., [54, 64]).

Another one of these clades is the Choristodera, a lineage of diapsid reptiles with a fossil record reaching back deep into the first half of the Mesozoic [56]. Choristoderes have been notoriously difficult to place on the phylogenetic tree of diapsids. They have a peculiar suite of anatomical features, including the presence of a neomorphic bone in the braincase [15, 27, 30], a complete set of palatal tooth plates [57], an elongated, flattened postorbital skull with expanded temporal fenestrae (e.g., [15, 19, 56]), and oval fenestrae on the ventral surface of the skull alongside the parasphenoid [15, 70]. Choristoderes are morphologically diverse, and include large, longirostrine forms exceeding 2 m in length [19], diminutive species with ornamented posterior skull roofs and generalized body plans [61], and long-necked, short-snouted species [25, 32].

Choristoderes were most diverse in the Jurassic-Cretaceous of Asia [6, 31, 32, 50, 56, 61, 77]. At the end of the Mesozoic, choristoderes seem to have experienced a major reduction in their diversity. Only three choristoderan genera are described from the Cenozoic: *Champsosaurus, Simoedosaurus*—members of the Neochoristodera—and the non-neochoristodere *Lazarussuchus* (e.g., [19, 20, 24, 56, 60]). The last of these, *Lazarussuchus*, finally became extinct during the Miocene [24].

The Paleocene of North America appears to have been a hotspot of choristodere diversity. At least four valid species of *Champsosaurus* and one species assigned to *Simoedosaurus* (*S. dakotensis*) are known from the Paleocene of the continent [15, 19, 20]. All of these species are among the largest choristoderes known and greatly exceed nearly all Mesozoic forms in size (e.g., [19, 20, 56]). In particular, *Simoedosaurus dakotensis* from the Paleocene of North Dakota, USA seems to have reached lengths of 3–4 m [20], making it one of the largest predatory amphibious reptiles of the Cenozoic apart from the largest crocodylians and snakes [1, 46]. Along with the type species of *Simoedosaurus*, *S. lemoinei* [35] from the Paleocene of France, *S. dakotensis* shows that at least one group of neochoristoderes departed from the extremely longirostrine skull anatomy exemplified by the giant longirostrine species *Champsosaurus gigas* from the Paleocene of Montana [19]. These records implicate choristoderes as an important lineage of large predators during the period of ecosystem reconstruction that took place in the first 5–10 million years following the K-Pg boundary.

The small amount of attention historically given to choristodere faunas has meant that the diversity of this clade in the Paleocene-Eocene of North America remains overlooked. Over the past century, several researchers have remarked on the existence of unrecognized choristodere species in collections from the major early Cenozoic vertebrate sites of the western United States [2, 20]. Although the majority of this material consists of isolated or associated postcrania [75], a number of nearly complete specimens remain undescribed.

In this contribution, I report on two new neochoristoderes from the Paleocene Polecat Bench Formation of Wyoming, USA. The two new species are based on exceptionally-preserved skulls and skeletons that allow for the observation of key regions, including the palate and braincase, in great detail. The two new species are dramatically different in skull form. One shows the most extreme example of posterior skull expansion and rostral robustification among choristoderes, whereas the other-a new species of *Champsosaurus*-possesses the longirostrine condition of that taxon. Key features differentiate both new species from other Cenozoic forms and strongly support the existence of two lineages of neochoristoderes in the same ecosystem. Along with a large species from North Dakota [20], the new brevirostrine taxon forms a clade of large North American neochoristoderes to the exclusion of European *Simoedosaurus*, suggesting biogeographic distinctions exist among short-snouted choristoderes. This implies an unexpected degree of taxic diversity among surviving choristoderes in the wake of the K-Pg and cautions against the referral of choristodere material from the same Cenozoic units to the same, previously described species.

### Geological setting

The holotype skeleton of the new champsosaurid was recovered from the Silver Coulee beds horizon of the Polecat Bench Formation at Fritz Quarry in Park County, Wyoming in 1954. The skull and postcrania of the holotype of the new simoedosaurid were recovered from the same formation 0.4 km northeast of Big Sand Coulee in 1968. The referred skeleton of the new simoedosaurid was recovered nearby in Park County during a 1964 expedition. Both specimens of the new simoedosaurid were reported in brief by Sigogneau-Russell and Donald [75]. All three choristodere skeletons reported in this contribution were collected by Princeton University Wyoming expedition crews. The Polecat Bench Formation is a Late Paleocene (late Tiffanian; [38]) unit that crops out in southern Wyoming in the Bighorn Basin and contains a rich fauna of lizards and mammals that has been documented since the mid-twentieth century [36, 37, 45, 48, 49]. The formation overlies the Cretaceous Lance Formation and underlies the Eocene Willwood Formation. In this region, it consists of gray claystone beds interspersed with lignite facies [45].

All three specimens consist of skulls and partial skeletons recovered in partial articulation. Skulls were found completely articulated. The skulls of the holotype and referred specimen of the new simoedosaurid are still partially attached to grey/tan claystone matrix, as are several articulated vertebrae in the referred specimen. All three skulls are in excellent condition, preserving most of the sutural connections between the individual cranial bones. Although the holotype of the new champsosaurid is affected by several fractures perpendicular to the main axis of the skull, the braincase and posterior skull remain in excellent condition.

### Systematic paleontology

Reptilia Laurenti 1768

Diapsida Osborn 1903

Choristodera Cope 1884b

Neochoristodera [23]

Simoedosauridae Lemone 1884

*Kosmodraco* gen. nov.

**LSID** urn:lsid:zoobank.org:act:84D898B5-57DB-4D91-9714-22BCB0B1D06E.

**Etymology** Greek κοσμος (ornamented) + Latin draco (dragon), referring to the ornamented posterior cranial bones of this genus.

**Diagnosis** Simoedosaurids with proportionally shortest rostrum among neochoristoderes (30–33% of skull length, compared with ~ 50% in *Simoedosaurus lemoinei*; [74]); triangular skull nearly equilateral (isosceles in *S. lemoinei*, which also possesses a more longirostrine preorbital skull; [74]; absence of ventral bifurcation of anterior margin of rostrum (present in *S. lemoinei*; [74]); narial opening posteriorly divided in dorsal view by anterior process of nasal, which forms an incomplete nasal bar (absent in *S. lemoinei*; [74]); lateral margins of postorbital skull confluent with anterior skull, such that the lateral margins of the skull are straight to convex (rather than strongly concave as in *S. lemoinei*; [74]) in dorsal view; orbits mediolaterally wider than anteroposteriorly long (opposite in *S. lemoinei*; [74]); postorbitofrontals with subrectangular main body and posteriorly directed squamosal flange (subtriangular main body and posterolaterally oriented squamosal flange in *S. lemoinei*; [74]); infratemporal and postorbital fenestrae equal in width (infratemporal fenestra more than twice as wide as the postorbital fenestra in *S. lemoinei*; [74]); nodular squamosal ornamentation restricted to posterior margin (nodules extend as rows along postorbitofrontal process of squamosal in *S. lemoinei*; [74]); vomerine tooth plates form less than one third of dentigerous palate; all palatal teeth blunt (posterior palatal teeth recurved in *Simoedosaurus*; [58]); mediolaterally expanded apices of dorsal and sacral neural spines (absent in *S. lemoinei*; [20]).

**Type species**: *K. dakotensis.*

**Remarks** I include the two brevirostrine North American simoedosaurids in their own genus, rather than consider them a subclade within *Simoedosaurus*, for several reasons. Both the large number of morphological differences between *Kosmodraco* and *Simoedosaurus lemoinei* (see above) and the degree of geographic and temporal speciation between these lineages (trans-Atlantic separation for several million years, if not before the K-Pg extinction; Fig. 11) favor the recognition of two genera.

*K. magnicornis* sp. nov.

**LSID** urn:lsid:zoobank.org:act:C608A280-1CD7-4869-A14C-C59D0D618601.

**Etymology** Latin magnus (large) + cornum (horn), referring to the particularly large squamosal spikes found on this species.

**Holotype** YPM VPPU 19168, nearly complete articulated skull, portion of dentary, scapula, coracoid, and associated cranial and postcranial fragments.

**Referred Material** YPM VPPU 18724, articulated partial skull, both mandibles, 34 vertebrae in various states of articulation, and fragments.

**Fig. 1:**
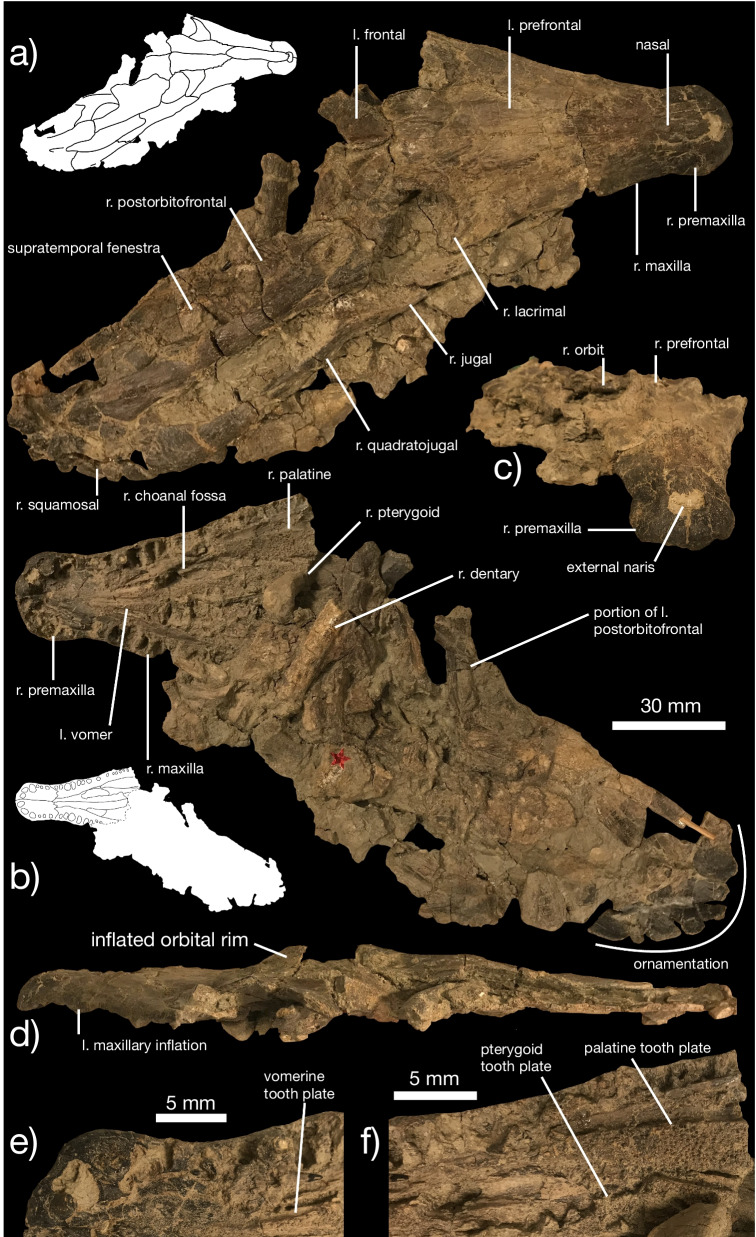
Cranial anatomy of *Kosmodraco magnicornis* gen. et sp. nov. Holotype skull YPM VPPU 19168 in **a** dorsal, **b** ventral, **c** anterior, and **d** right lateral views, with details of the palatal anatomy at the anterior end **e** and midway along **f** the skull. Inset black and white drawings illustrate borders between bones

**Fig. 2:**
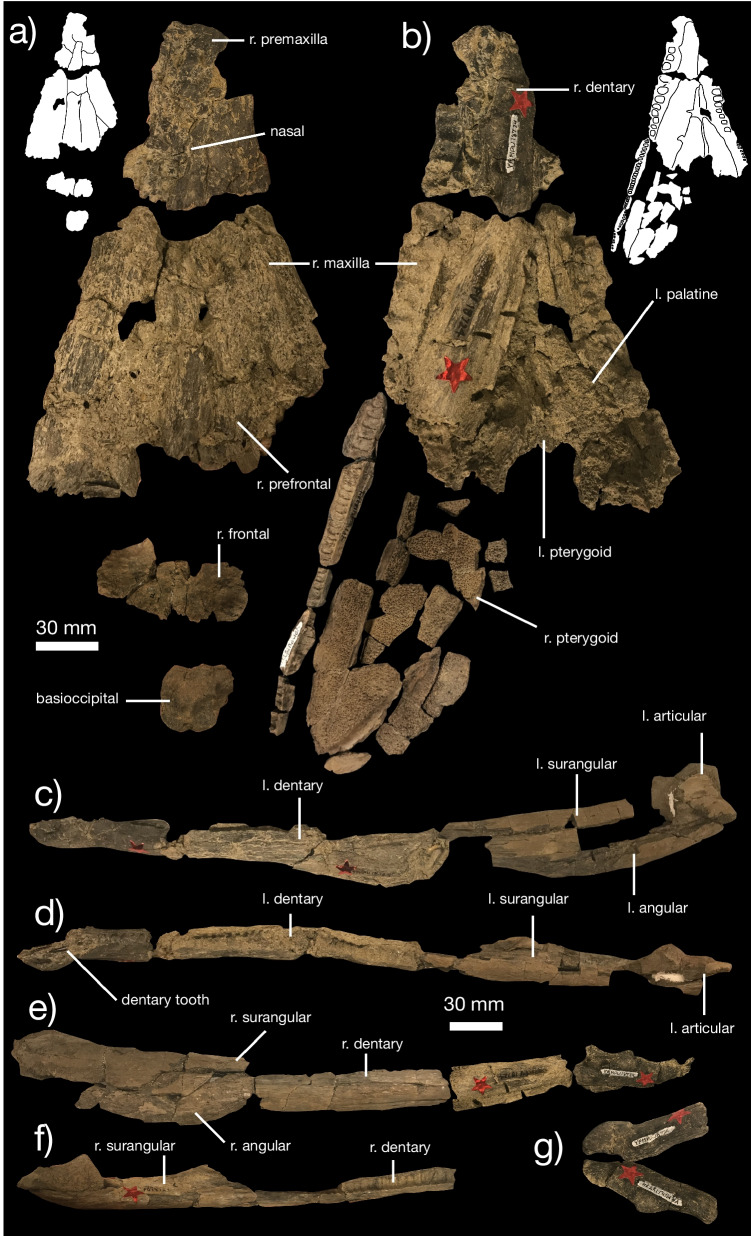
Cranial anatomy of *Kosmodraco magnicornis* gen. et sp. nov. Referred specimen YPM VPPU 18724 in **a** dorsal, and **b** ventral views, with complete left **c**, **d** and right mandibles **e**, **f** in **c**, **e** lateral and **d**, **f** dorsal views, and digitally articulated left and right dentary symphyses in **g** ventral view. Inset black and white drawings illustrate borders between bones

**Diagnosis** Reduction of mediolateral constriction between premaxillary and maxillary dental arcades in dorsal view (Figs. 1a, b, 2a, b; compared to a distinct constriction in *K. dakotensis*); squamosal posterior margin bears eight discrete posteriorly-projecting ornaments (Fig. 1a, b; three to four are present in *K. dakotensis* and *S. lemoinei*); quadratojugal ornamented with spurs like those present on squamosal (Fig. 1a; ornamentation restricted to squamosal in *K. dakotensis* and *S. lemoinei*); elongated subnarial fenestrae in palate exceeds more than half the length of the vomer (Fig. 1b; less than 33% in *K. dakotensis* and *S. lemoinei*); no diastema between premaxillary and maxillary teeth (Fig. 1b; tooth gap present in *K. dakotensis*); triangular vomer bears single ridge with two rows of teeth (Fig. 1b, e; vomer subovoid with at least three rows of teeth anteriorly and no defined ridge in *K. dakotensis*; [20, 58]); elongated infratemporal fenestra (5 × as anteroposteriorly long as wide; Fig. 1a; 3 × as long as wide in *K. dakotensis*); first three premaxillary alveoli greatly enlarged relative to rest of premaxillary and anterior maxillary alveoli (Fig. 1b, e; teeth gradually reduce in size along row in *K. dakotensis*); premaxilla with six teeth (four in *K. dakotensis*).

**Description** *Kosmodraco magnicornis* is a large-bodied neochoristodere (skull length = 431 mm) only exceeded in size by *K. dakotensis*, *Champsosaurus gigas*, and *Simoedosaurus lemoinei* among Choristodera [20, 56]. The holotype specimen includes a nearly complete skull, dentary fragment, a partial shoulder girdle, and additional fragments. The referred skull is less complete but clearly identifiable to the same species based on shared characters of the premaxilla-maxilla transition and premaxillary, maxillary, dentary, and palatal tooth morphology.

*Skull* The skull of *Kosmodraco magnicornis* (Figs. 1a–f, 2a–g) shares the blunt rostrum found in *S. lemoinei* [20, 57, 74] but particularly pronounced in *K. dakotensis* [20]. The lateral margins of the premaxillae are smoothly confluent with the rest of the skull (Figs. 1a, 2a), as in *Champsosaurus* spp. [15, 18, 19, 30] and most other neochoristoderes, including *Simoedosaurus lemoinei* [74], and *Liaoxisaurus chaoyangensis* [29]. The Cenozoic non-neochoristodere *Lazarussuchus inexpectatus* [47, 60] also possesses this condition. In *K. dakotensis*, the border of the premaxilla and maxilla is distinctly medially offset from the rest of the lateral margin of the rostrum [20]. Unlike *S. lemoiniei* (Fig. 2 in Sigogneau-Russell and Russell [74]), the anterior margin of the premaxillae does not appear slightly bifurcated in ventral view. This condition, shared with *K. dakotensis* [21], does not appear to be the result of wear sustained by this region of the skull, as the holotypes of both species of *Kosmodraco* have well-preserved anterior premaxillae. Ventrally, the premaxillae are smooth and form the anterior end of the palate. The first three premaxillary alveoli are more than twice as large as all other alveoli except two placed at the maxillary inflation. *K. dakotensis* lacks enlarged premaxillary alveoli, and instead shows an upper dental arcade with alveoli that gradually decrease in size [20].

The anterior margins of the fused nasals in *Kosmodraco magnicornis* form the posterior border of the external naris in dorsal view. Several short processes extend from the nasal into the naris, forming a very small internasal bar (Fig. 1a, c). Internasal bars are found in *Champsosaurus* spp. [9, 15, 18, 19, 30] and *K. dakotensis* [20, 60] supplementary codings [60]). However, *S. lemoinei* lacks a distinct process projecting anteriorly into the external naris [74]. The nasals are fused into a single element as in all other Cenozoic choristoderes except *Lazarussuchus* [60], which possesses the plesiomorphic condition of paired nasals found in Mesozoic choristoderes such as *Coeruleodraco jurassicus* [61], *Philydrosaurus proseilus* [29], and *Monjurosuchus splendens* [28]. Together with the premaxillae and anterior maxillae, the anterior nasals are heavily ornamented with various ridges and sulci (Fig. 1a, c). This is similar to the condition in *K. dakotensis* [20] and other large Cenozoic neochoristoderes [19, 30, 74]. The nasal forms tight sutural connections with both maxillae, premaxillae, and prefrontals (Additional file 1).

**Fig. 3:**
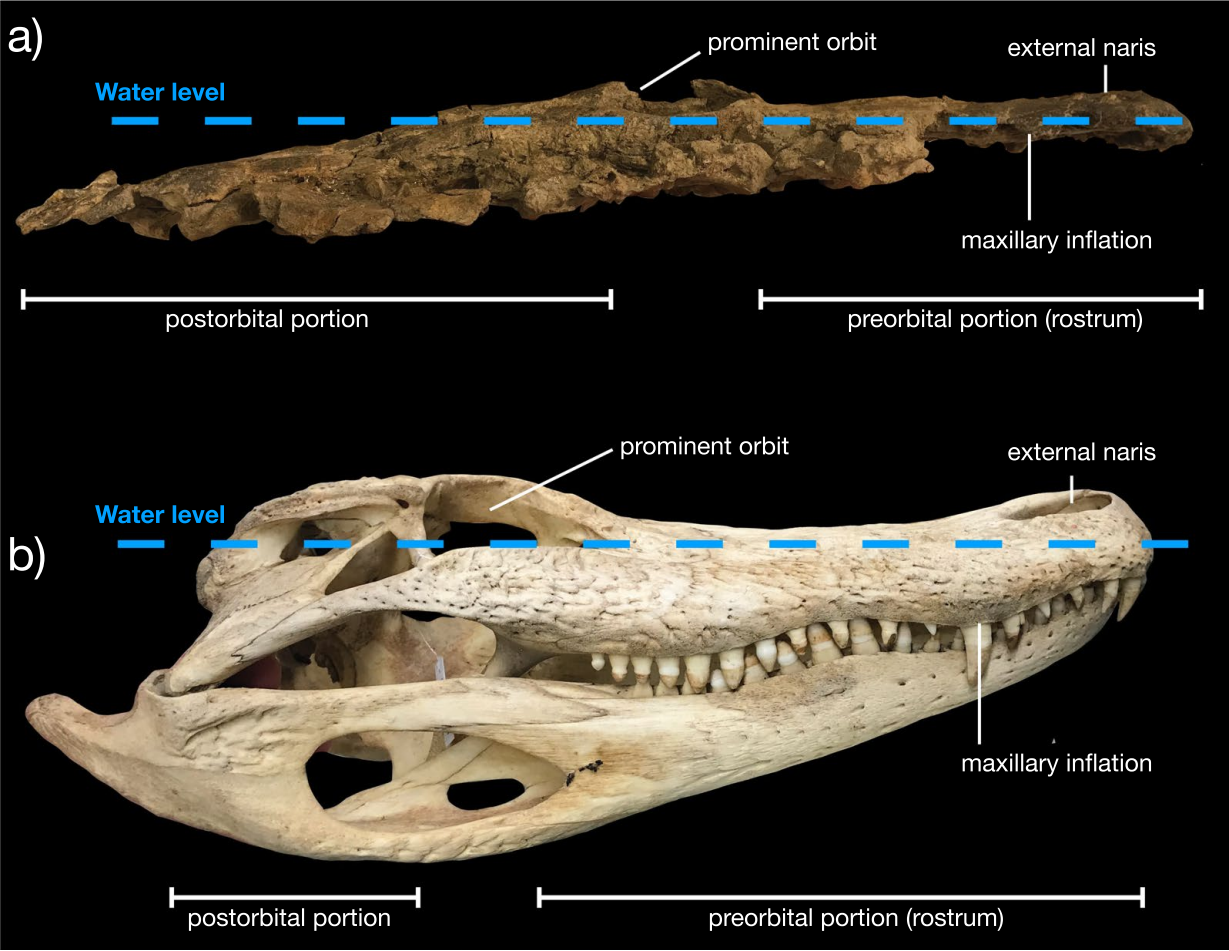
Lateral cranial profile of *Kosmodraco magnicornis* gen. et sp. nov. and *Alligator mississippiensis* compared. Skulls of *K. magnicornis* (**a**) and *A. mississippiensis* (**b**) in right lateral view (not to scale)

The maxillae form the majority of the rostrum in *Kosmodraco magnicornis* (Figs. 1a, b, 2a, b). The maxillae are highly ornamented bones. However, the maxillae lack any trace of enlarged neurovascular foramina like those found in crocodyliforms (Fig. 3b). The maxilla articulates with the premaxilla anteriorly, the nasal laterally, and the jugal and lacrimal posteriorly. Ventrally, the maxilla bears at least 31 tooth positions (Figs. 1b, 2b). Approximately one third of the way along the anteroposterior run of the maxilla, the bone is ventrally inflated. This inflation corresponds to two enlarged alveoli and is comparable to the condition in modern crocodyliforms (e.g., *Alligator mississippiensis*; Fig. 3b). This inflation is also present in *K. dakotensis*, but it is reduced and placed far anteriorly to border the suture with the premaxilla [20]. Posterior to the maxillary inflation, alveoli gradually decrease in size. The maxillary tooth row terminates below the orbit (Additional file 2).

The prefrontals of *Kosmodraco magnicornis* are elongated, subtriangular, and bear a rugose surface texture consisting of meandering ridges. These bones articulate with the maxillae and lacrimals via clearly visible tight interdigitating sutures. The lacrimals are similar to those in *Simoedosaurus lemoinei* [74], *Champsosaurus* spp. [9, 19, 30], and *Tchoiria klauseni* [50], but are mediolaterally widened relative to the condition in *K. dakotensis* [20]. These bones contribute to the relatively greater mediolateral expansion of the posterior skull in *K. magnicornis* relative to *K. dakotensis*. The frontals are weakly ornamented bones that divide the orbitals medially. They are intermediate in width between the condition in *Champsosaurus* spp. and *S. lemoinei* [9, 15, 20, 30, 73, 74]). The orbits are subcircular and surrounded by raised regions of rugose bone. These produce a raised appearance for the orbits in lateral and medial view that resembles the condition in surface-cruising amphibious tetrapods, including crocodylians (Fig. 3). The raised orbits are apparently more prominent in *K. magnicornis* than the holotype of *K. dakotensis*, but the latter skull is distorted from crushing [20] and thus this feature cannot be used to distinguish these species.

The preserved left side of the posterior end of the skull in YPM VPPU 19168 includes a postorbitofrontal that medially bounds the anterior two-thirds of the infratemporal fenestra (Fig. 1a). The shape of this bone is extremely similar to the condition in *Kosmodraco dakotensis*, where the main body is rectangular and the squamosal flange is directed posteriorly parallel to the main axis of the skull. This flange is directed posterolaterally in *Simoedosaurus lemoinei* [56, 57, 74]. Posteriorly, the postorbitofrontal contacts the triradiate squamosal. The squamosal bears eight distinct nodule-like processes along its posterior margin. Similar ornamentation has been reported in *K. dakotensis* [20] and *Champsosaurus* [15]. However, the development of these ornaments in *K. magnicornis* is greater than in any other Cenozoic [19, 20, 73] or late Mesozoic [50] neochoristodere. Instead, the ornamentation in *K. magnicornis* approximates or exceeds the squamosal ornamentation in Jurassic and Early Cretaceous choristoderes like *Coeruleodraco jurassicus* [61] and *Monjurosuchus splendens* [28]. Unlike *S. lemoinei* [74], *C. jurassicus* [61], or *M. splendens* [28], the postorbitofrontal process of the squamosal lacks any ornamentation in *K. magnicornis* (Fig. 1a). This condition is shared with *K. dakotensis* [20]. The quadratojugal flange of the squamosal sharply curves anteromedially to contact the quadratojugal, forming a corner at the posterior end of the infratemporal fenestra. Unlike *K. dakotensis* [20] or *S. lemoinei* [74], the external margin of the quadratojugal in *K. magnicornis* bears at least one projecting ornament closely comparable to those found on the squamosal. The dorsal surface of the quadratojugal lacks ornamentation.

Laterally, the anterior half of the infratemporal fenestra in *Kosmodraco magnicornis* is bounded by the jugal, which is laterally straight to convex as in *K. dakotensis* [20] but unlike the concave condition in *S. lemoinei* [73]. The jugal and quadratojugal are slightly mediolaterally widened relative to the slender bones in *K. dakotensis* [20] *Champsosaurus* spp. [9, 15, 19, 30], and *Tchoiria klauseni* [50].

Ventrally, the holotype and referred skull preserve nearly complete palates and tooth rows (Figs. 1b, 2a). Premaxillary and maxillary dentition consists of striated, conical crowns with sub-thecodont implantation, as in other neochoristoderes [9, 19, 20, 30, 74]. The three anterior premaxillary alveoli bear enlarged fangs, a condition shared with *S. lemoinei* that contrasts with the morphology found in *Kosmodraco dakotensis* [20], *Champsosaurus* spp. [19], *Tchoiria klauseni* [50], and non-neochoristoderes (e.g., [28, 60, 61]). Premaxillary, maxillary, and dentary teeth lack serrations.

The preserved palate in the holotype and referred skulls of *Kosmodraco magnicornis* includes the premaxillae, vomers, palatines, and pterygoids (Figs. 1b, e, f; 2b). The vomers each bear a midline ridge and form the anterior half of the medial margins of the elongated choanal fenestrae. The vomer tapers anteriorly such that this bone has a triangular outline (Fig. 1e); in *K. dakotensis*, the anterior vomer is subovoid and bears at least three rows of teeth anteriorly (Fig. 9 in [58]). The vomers project anteriorly to the level of the premaxilla-maxilla suture. The vomer articulates with the pterygoid posteriorly and forms much of the medial border of the choanal fossa laterally. The choanal fossa is enlarged relative to those in *K. dakotensis* [20] and *Simoedosaurus lemoinei* [74], and approaches the condition in *Tchoiria klauseni* [50].

In *Kosmodraco magnicornis*, the choanal fossa posteriorly grades into the nasopalatal trough, which separates the two major tooth-bearing plates of the posterior palate (Fig. 1b). These are formed by the medial tooth plate of the pterygoid and the combined palatine and lateral pterygoid tooth plate, respectively, matching the condition in *K. dakotensis* and other neochoristoderes [58]. The pterygoid and palatine are significantly wider than the vomer, a condition shared with *Tchoiria klauseni* [50], *Simoedosaurus lemoinei* [74], *K. dakotensis* [20], and *Ikechosaurus sunalinae* [58] among neochoristoderes. The vomerine and palatal tooth plates are composed entirely of small, vertically oriented, blunt teeth as in *K. dakotensis*, but unlike European *Simoedosaurus* where posterior palatal teeth are recurved [57].

Posterior to the bony palate, the ventral surface of the holotype skull is more poorly preserved (Fig. 1b). Several bones are potentially identifiable, but they are too damaged to characterize. The referred specimen includes a basioccipital that bears a sub-ovoid, slightly bifurcated occipital condyle as in *K. dakotensis* [20] *Champsosaurus* spp. [9, 15, 30], and *Tchoiria klauseni* [50].

*Mandible* The lower jaw is represented in YPM VPPU 19168 by a fragment of the elongated dentary and in YPM VPPU 18274 by both nearly complete mandibles (Figs. 1b, 2). The dentary is an elongated, dorsoventrally shallow element. Anteriorly, the symphysis of each dentary is mediolaterally expanded, forming a broad symphyseal region (Fig. 2a, c, d). This feature differentiates *Kosmodraco magnicornis* and *Simoedosaurus lemoinei* from *Champsosaurus* spp., which possesses a minimally expanded dentary symphyseal region [75]. The mandible as a whole is robust relative to the extremely elongated set of bones in *Champsosaurus* spp. [18, 19, 30]. 40 tooth positions are present in the complete left dentary of YPM VPPU 18274. Alveoli maintain relatively constant size along the anteroposterior axis of the dentary and terminate at the level of the orbit. Unlike *Champsosaurus* spp. [18], the dentary is not distinctly downturned relative to the posterior mandibular bones. The surangular, angular, and articular comprise approximately 30% of the length of the mandible. Medially, the surangular bears a shallow fossa. The retroarticular process is small and rounded. As in the premaxilla and maxilla, dentition consists of elongated, conical teeth with apicobasally-running striations and slight mesiodistal curvature. This type of dentition is characteristic of neochoristoderes (e.g., [30]).

**Fig. 4:**
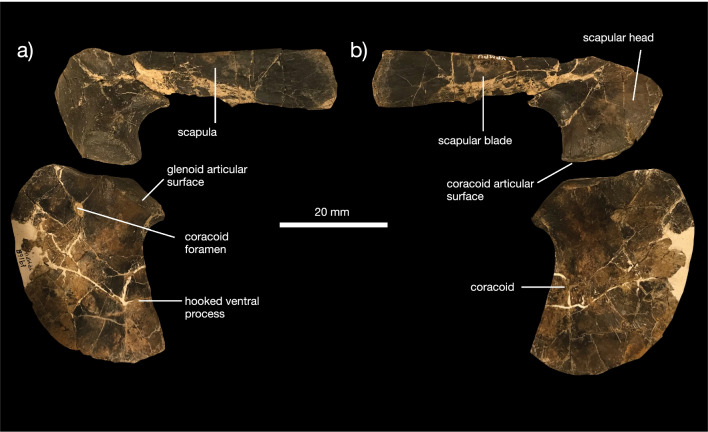
Postcranial anatomy of *Kosmodraco magnicornis* gen. et sp. nov. Left shoulder girdle of *K. magnicornis* holotype YPM VPPU 19168 in **a** lateral and **b** medial views

*Shoulder girdle *The shoulder girdle of *K. magnicornis* is represented by the unfused scapula and coracoid included in the holotype YPM VPPU 19168 (Fig. 4a, b). The scapular head is large and ventrally directed relative to the straightened scapular blade. The acromion process is weakly developed. The coracoid articular surface is large and appears posteriorly hooked, unlike the condition in *Champsosaurus* [20]. The scapular blade is proportionately longer than the blade in *Champsosaurus* [20]. The coracoid is a large, plate-like bone with a concave scapular articular facet, a small coracoid foramen, and a large, hooked coracoid process.

**Fig. 5:**
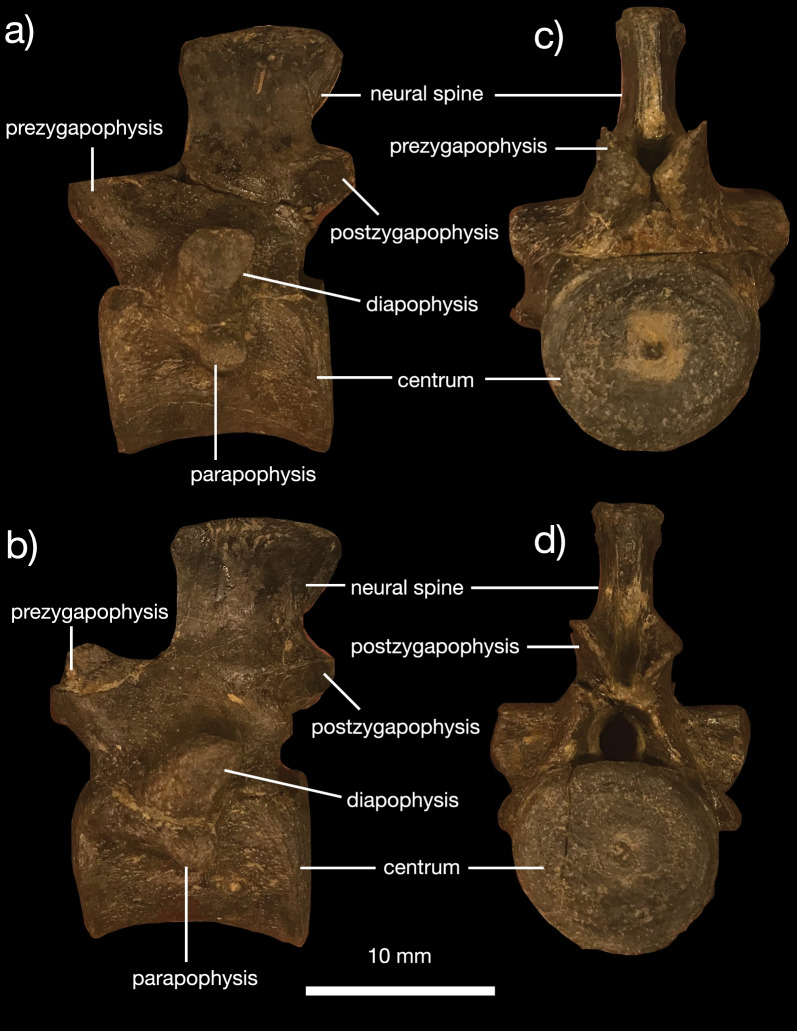
Postcranial anatomy of *Kosmodraco magnicornis* gen. et sp. nov. Dorsal vertebrae of referred specimen in **a**, **c** left lateral and **b**, **d** anterior views

**Fig. 6:**
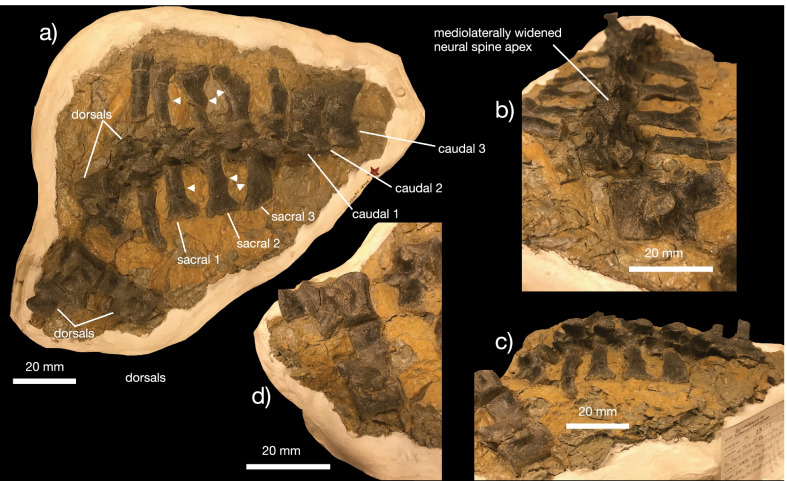
Postcranial anatomy of *Kosmodraco magnicornis* gen. et sp. nov. Semi-articulated posterior dorsals, sacrum, and anterior caudals of referred specimen in **a** dorsal, **b** anterior, and **c** lateral views, with **d**, detail of the disarticulated dorsals still embedded in the block. Arrows point to rugosities on sacral ribs for soft tissue attachment

**Fig. 7:**
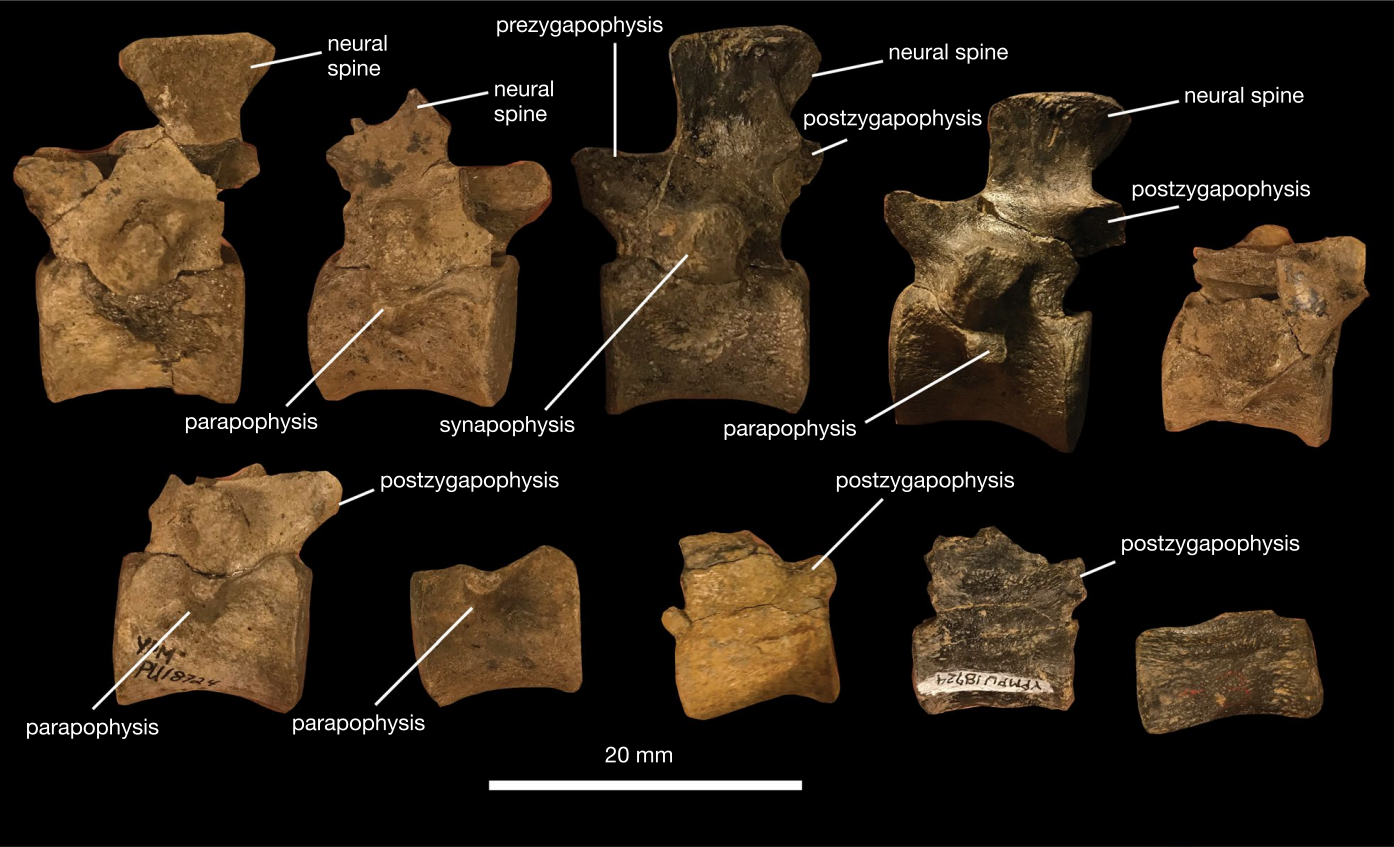
Postcranial anatomy of *Kosmodraco magnicornis* gen. et sp. nov. Selected well-preserved caudal vertebrae of referred specimen in lateral view

*Vertebral column *The referred specimen includes 34 complete vertebrae, including an exquisitely preserved, articulated series from the sacral region and tail base (Figs. 5, 6, 7). This region of the skeleton compares closely with the holotype of *Kosmodraco dakotensis* [20]. The dorsal and sacral vertebrae are closely comparable in morphology: the neural spines are short, rectangular and mediolaterally expanded at their rugose apices, the synapophyses are prominent, and the centra are amphiplaytan (Figs. 5a, b, 6). The pronounced mediolateral expansion of the dorsal apices of the neural spines serve to distinguish *Kosmodraco* from *Simoedosaurus* [20]. As in other neochoristoderes, the neurocentral sutures are not fully fused and there are slight depressions between the synapophyses and parapophyses in the dorsal and caudal vertebrae (e.g., [18–20]).

The number of sacrals in *K. magnicornis* is the same as in *K. dakotensis* and all other choristoderes except *Lazarussuchus* spp., which has four sacrals [60]. As in *K. dakotensis* and other neochoristoderes, the sacral ribs are widened relative to adjacent dorsal and caudal ribs. The sacral ribs also show pronounced, symmetrical bulges for attachment of the pelvic musculature as in extant crocodylians (e.g., [12; 69; 71]), and the ribs of the first and third sacrals are curved towards the central to form a bony plate with the ribs of sacral 2 (Fig. 5a) as in *Lazarussuchus* sp. [60], but do not overlap as in *Champsosaurus* spp. [20]. The ribs of sacrals 2 and 3 in *K. magnicornis* differ from those in *K. dakotensis* in possessing developed protuberances placed midway along their posterior surfaces. The ancestral diapsid condition is two sacrals, which is conserved in all crocodylians except some giant Miocene caimans [71]. The ribs of the last dorsal vertebra and first caudal lack these protuberances. These protuberances are associated with a pronounced set of lineations running approximately parallel to the mediolateral axis of the sacrum. The sacrals are unfused, and faint suture separate all sacral ribs from their corresponding vertebrae. As in *K. dakotensis*, there is a consistent but minute degree of asymmetry in the posterior dorsals and sacrals, although this may be due to post-mortem deformation.

The caudal series included in the referred specimen of *K. magnicornis* consists of 25 preserved vertebrae, thirteen of which are mostly complete (Figs. 6, 7). These are again closely similar to the caudals of *K. dakotensis*: anterior caudals are essentially identical in morphology to the sacrals, and the caudals possess neural spines that become thinner towards the posterior end of the series [20]. The first caudal has mediolaterally widened ribs that curve slightly anteriorly towards the sacrum. Posteriorly, the sacral ribs shorten. There is no clear longitudinal ridging on the caudal centra in YPM VPPU 18274 as in the holotype of *K. dakotensis* [20].

Reptilia Laurenti 1768

Diapsida Osborn 1903

Choristodera Cope 1884b

Neochoristodera [23]

Champsosauridae Cope 1884

*Champsosaurus* Cope 1876

*C. norelli* sp. nov.

**LSID** urn:lsid:zoobank.org:act:D2F2CE49-CD9F-44FA-8F45-78D70C68E638.

**Etymology** *Norelli*, after Mark Norell, curator of vertebrate paleontology at the American Museum of Natural History, for his extensive contributions to tetrapod paleontology and evolution.

**Holotype** YPM VPPU 16511, complete skull, mandibles, and partial skeleton.

**Diagnosis** Differs from other species of *Champsosaurus* in the following combination of features: relatively short snout (shared with *C. natator*; differs from *C. gigas*, *C. tenuis*, *C. lindoei*, *C. ambulator*, *C. laramiensis*, and cf. *C. albertensis*; [9, 18, 19, 22, 30], mediolaterally unexpanded rostrum (shared with *C. gigas* and *C. laramiensis*; [9, 18, 19], strongly ventrally deflected postorbital skull in lateral view; mandible strongly arched, such that it is concave ventrally and convex dorsally (shared with *C. gigas*; [19]), parietal table is not bifurcated (differs from *C. gigas*, *C. natator*, *C. lindoei*, *C. natator*, and *C. albertensis*); paroccipital processes formed by opisthotic, neomorphic bone, and posterior process of the pterygoid abruptly deflected laterally at level of occipital condyle.

**Remarks** *Champsosaurus norelli* sp. nov. is assignable to *Champsosaurus* based on the presence of the following characters [19, 30]: extreme longirostry,interorbital width exceeded by orbital width; reduced lacrimal; premaxilla and vomer do not contact; presence of an internarial bar; craniomandibular joint positioned anterior to occipital condyle; shortened suborbital fenestra; ventral deflection of paroccipital process; basal tubera elongated; dentary symphysis extends more than halfway along tooth row; splenial participates in mandibular symphysis.

**Fig. 8:**
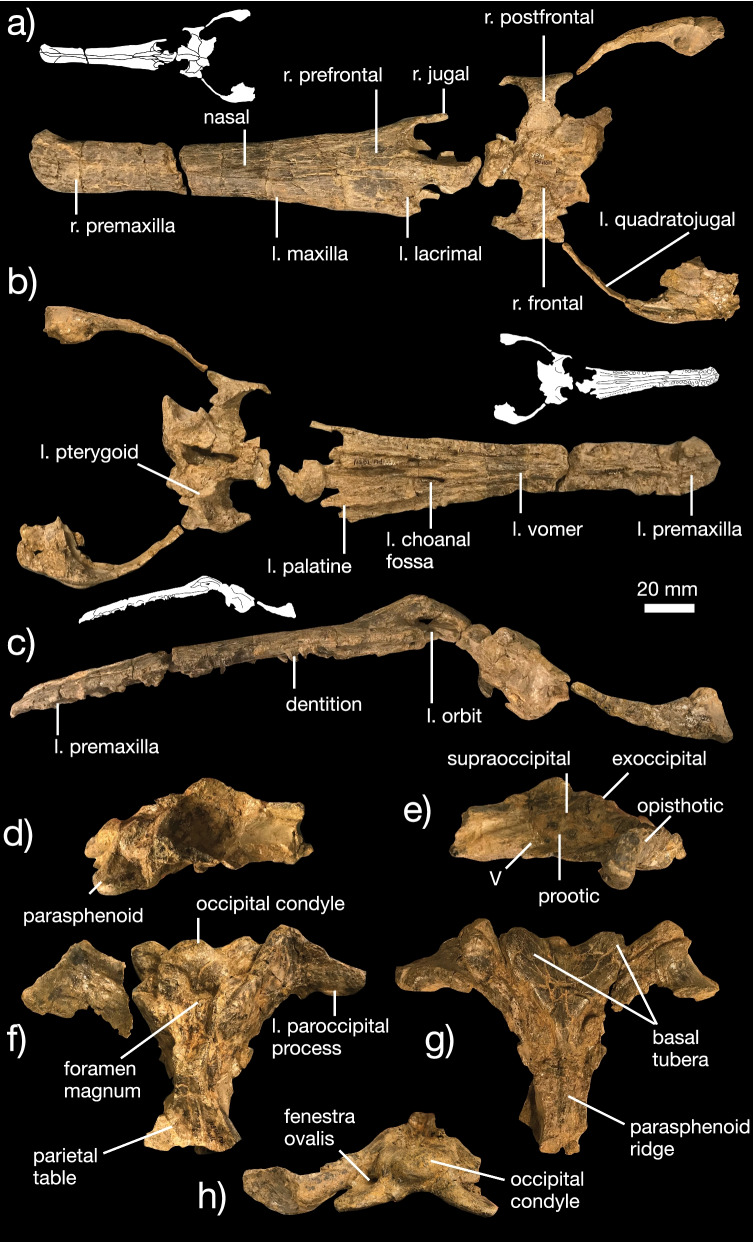
Cranial anatomy of *Champsosaurus norelli* gen. et sp. nov. Skull in dorsal (**a**), ventral (**b**), and left lateral (**c**) views. Braincase in **d** right lateral, **e** left lateral, **f** dorsal, **g** ventral, and **h** posterior views. Inset black and white drawings illustrate borders between bones

**Description** *Champsosaurus norelli* is a longirostrine neochoristodere (Fig. 8a–c) resembling *Champsosaurus* spp.*, Ikechosaurus gaoi*, *Mengshanosaurus minimus*, and *Tchoiria klauseni* (e.g., [15, 19, 22, 30, 50, 82]). It is a large neochoristodere, resembling in size the Fort Union Formation *C. gigas* specimen YPM VPPU 16240 [19]. This places *C. norelli* in the upper size bracket for choristoderes, along with most other Cenozoic North American taxa (i.e., *C. gigas*, *Kosmodraco* spp.; [18–20, 30]).

*Skull *Although abrasion of the external surface of the rostrum makes it difficult to describe individual elements in detail, it is clear that the nasals are fused, the premaxillae are restricted to the anteriormost portion of the skull, and the anterior rostrum is slightly mediolaterally expanded. The longirostrine condition in *C. norelli* is not as pronounced as is most species of *Champsosaurus*, including *C. gigas* [19], *C. lindoei* [15, 30], *C. tenuis* [22], *C. ambulator* [9], *C. tenuis* [22], and *C. laramiensis* [9]. There is no strong mediolateral expansion of the rostrum at the level of the premaxilla, differentiating *C. norelli* from all other species of *Champsosaurus* besides *C. gigas* and *C. laramiensis* [9, 18, 19].

The lacrimals are reduced as in *Champsosaurus* spp. [15, 30], and the prefrontals are elongated. The orbits appear particularly long, measuring at least twice as long anteroposteriorly as wide mediolaterally and constituting at approximately one fifth of the total length of the skull. However, it is unclear if this feature is affected by erosion, as there is a break in the skull approximately halfway along the run of the orbits. The orbits are strongly emarginated by the frontals, lacrimals, and jugals. In lateral view, the orbits are clearly raised. As in *Champsosaurus* spp., the frontals are pinched between the orbits to form an extremely small interorbital bar [15, 30].

The postorbital and postfrontal are unfused in *C. norelli*, unlike the condition in *Kosmodraco dakotensis* or *K. magnicornis*. Unlike *Simoedosaurus lemoinei* [74] and most species of *Champsosaurus* [15, 19, 22], but similar to *Tchoiria* [50] and Mesozoic choristoderes (e.g., [61, 82]), the postorbital region of the skull is relatively uninflated in *C. norelli*. This region is less than one third wider mediolaterally than the skull is at the level of the orbits. The external margin of the infratemporal fenestra is formed by the fused jugal and quadratojugal, which are particularly thin in *C. norelli*. A similarly thin lateral border of the infratemporal fenestra is found in some species of *Champsosaurus* (e.g., *C. laramiensis*, *C. lindoei*, [9, 15, 18, 19, 30]). *Tchoiria klauseni*, in contrast, possesses a widened infratemporal fenestra border [50]. The condition in *K. dakotensis* and *K. magnicornis* is intermediate between the extremes represented by *C. norelli* and *T. klauseni* (Fig. 1, [20]). Unlike both species of *Kosmodraco* and some species of *Champsosaurus* (e.g., *C. lindoei*, [15, 30]), the quadratojugal and squamosal are unornamented in *C. norelli.*

Medially, the squamosal extends to contact the posterior end of the parietal table (Fig. 8a). The parietal of *C. norelli* is posteriorly widened and dorsally bears a Y-shaped table that forms the medial margin of the supratemporal fenestra with the greatest concavity anteriorly. The shape of the parietal table shows a consistent degree of interspecific variation in neochoristoderes. In *Champsosaurus gigas*, the table is small and U-shaped, with the concave side facing posteriorly [19]. In *C. lindoei*, two distinct ridges run approximately parallel to the long axis of the skull and are anteriorly broken up into distinctive nodules [15]. Two parallel ridges are also present in *C. natator* [15] Fig. 1) and *C. laramiensis* [9]. In *C. ambulator*, these ridges meet midway alone the parietal, forming an x-shaped table. Both species of *Kosmodraco* show little development of the parietal table [20], Fig. 1a).

Ventrally, the premaxilla and maxilla bear approximately 34 teeth (Fig. 8b, c). These show the classic conical, striated form of neochoristoderes. The vomers are tooth-bearing elements that each possess a single palatal tooth row anteriorly and two posteriorly. The elliptical choanal fossae are also placed midway along the palate, unlike the posteriorly retracted condition present in other species of *Champsosaurus* [15, 30]. The palatines emerge lateral to the vomers at the level of alveolus 17. Each bears a single row of palatal teeth and posteriorly bifurcates to form the borders of the choanal fossa. The pterygoids are the widest tooth-bearing palatal bones and extend posteriorly to meet the braincase and posterolaterally to meet the ectopterygoids. Unfortunately, a large fracture in the ventral portion of the skull at the level of the orbits makes it impossible to describe the precise morphology of these articulations.

The braincase is excellently preserved (Fig. 8a, b, d–h). The parasphenoid is hourglass-shaped in ventral view and is fused to the basisphenoid dorsally and the basioccipital ventrally (Fig. 8f, g). The median pharyngeal recess is well-developed, as in *Champsosaurus lindoei* [15]. Posteriorly, the parasphenoid forms the bases of the basal tubera, which are reduced relative to *C. lindoei* [15], *C. laramiensis* [9], and *C. gigas* [19] but similar in size to those in *K. dakotensis* [20]. The parasphenoid differs from those in *Champsosaurus* spp. [9, 15] and *K. dakotensis* [20] in bearing an anteroposteriorly-running ridge that develops from the lateral margins of the medial pharyngeal recess. The laterally directed processes formed by the posterior flange of the pterygoid, opisthotic, and neomorphic bone is strongly angled at the level of the apex of the posterior process of the opisthotic, producing a distinct ‘kinked’ shape that is not found in any other species of *Champsosaurus* [9, 15] or *K. dakotensis* [20]. The supraoccipital forms the dorsal wall of the posterior half of the braincase and is slightly concave ventrally as viewed in posterior view (vs. strongly concave in *C. lindoei*; [15]). The lateral contact with the prootic, opisthotic, and exoccipital (Fig. 8d, e) is typical of choristoderes [15]. The exoccipital defines the ventral surface of the braincase (Fig. 8g–h) and posteriorly terminates in an ovoid occipital condyle. The occipital condyle is not slightly bifurcated as in *C. lindoei* [15], *C. ambulator* [9], *C. gigas* [19], or *K. dakotensis* [20], but comparable to the condition in *C. laramiensis* [9].

**Fig. 9:**
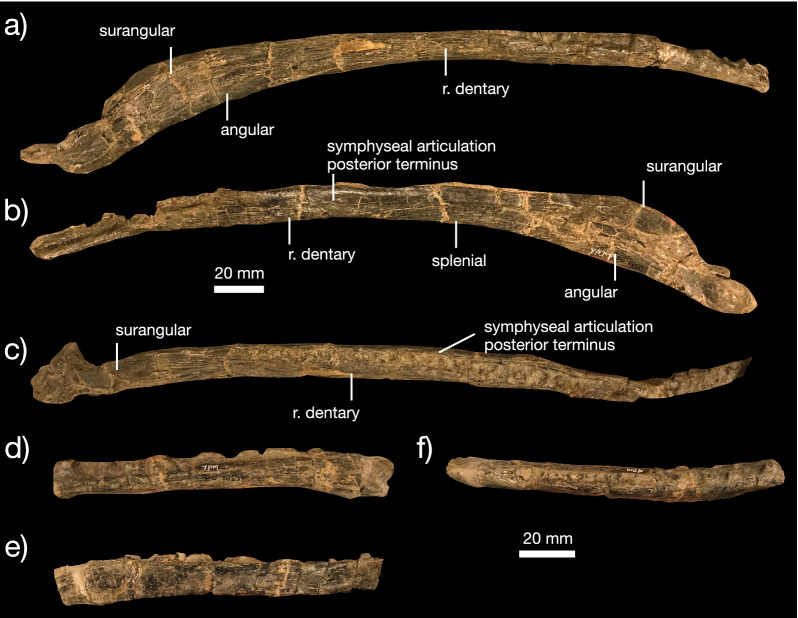
Mandibular anatomy of *Champsosaurus norelli* gen. et sp. nov. Complete right mandible in **a** lateral, **b** medial, and **c** dorsal views. Partial left mandible in **d** medial, **e** lateral, and **f** dorsal views

*Mandible *The mandible of *C. norelli* includes an elongated, downturned dentary (Fig. 9a–f) as in *Champsosaurus gigas* [18] but unlike other species of *Champsosaurus* [9, 30]. The dentary bears at least 34 tooth positions, and the dentary alveoli are slightly smaller than those in the maxilla and premaxilla. Posteriorly, the dentary articulates with the angular ventrally and surangular dorsally. Due to abrasion of the bone surface, it was not possible to determine the precise morphology of these articulations. However, the groove on the medial surface of the splenial shows the apomorphic condition of *Champsosaurus* in which the splenial participates in the mandibular symphysis was present in *C. norelli* (Fig. 9b).

**Fig. 10:**
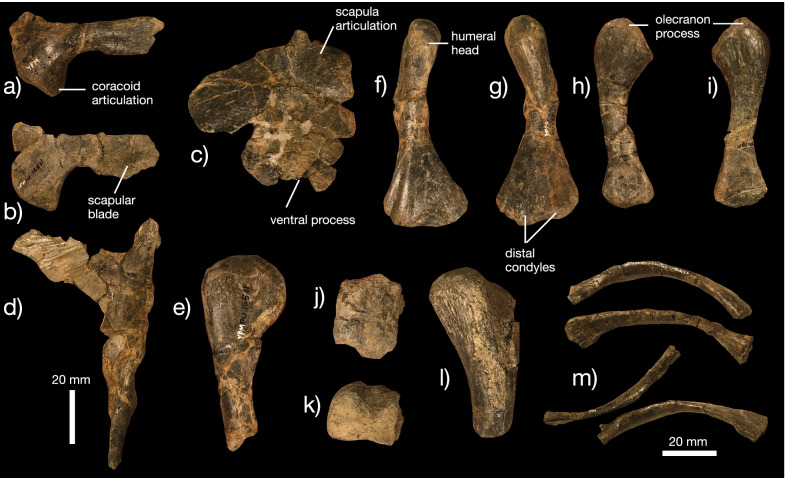
Postcranial anatomy of *Champsosaurus norelli* gen. et sp. nov. Left **a** and right **b** scapulae in **a** lateral and **b** medial views. Right coracoid in **c** medial view. Interclavicle in **d** anterior view. Proximal left humerus in **e** anterior view. Right humerus in **f** lateral and **g** medial views. Right ulna in **h** lateral and **i** medial views. Dorsal centrum in **j** dorsal and **k** lateral views. Proximal femur fragment in **l** anterior view. Ribs (**m**) of *C. norelli*

*Shoulder girdle* A nearly complete shoulder girdle, including the scapula, coracoid, and interclavicle, is known for the holotype of *Champsosaurus norelli* (Fig. 10a–d). The scapula possesses a dorsoventrally shallow blade relative to the condition in *Champsosaurus gigas* (Fig. 10a, b; [19]), but unlike the extremely shallow condition in *Kosmodraco magnicornis* (Fig. 4) or *Simoedosaurus lemoinei* [73]. The ventral expansion of the scapular head (Fig. 10a, b) is similar to *K. magnicornis* and *C. gigas*, but differs from other *Champsosaurus* species [19, 22, 30]. The coracoid is large and expansive. Unfortunately, abrasion of this bone means that beyond its general similarity to other choristodere coracoids, the anatomy of this element in *C. norelli* cannot be described (Fig. 10c). The interclavicle is broken but shows the strongly pointed posterior apex found in *C. laramiensis* [9]. The anterolateral processes are proportionately longer than those in *C. gigas* [19], but approximate the condition in *C. laramiensis* [9] and *C. tenuis* [22].

*Limbs *The humerus (Fig. 10e–g) is of a similar morphology to most other neochoristoderes, showing the prominent proximal and distal expansion found in other species of *Champsosaurus * (e.g., [9, 19]). Unlike *C. laramiensis* but similar to *C. gigas*, the proximal end of the humerus lacks a developed proximal endotuberosity [19]. The deltopectoral crest is weakly developed, as are both the distal condyles (unlike *C. laramiensis*, [19]). The distal endotuberosity is also poorly developed, contrasting with the condition in *C. laramiensis* [9].

The ulna is massively constructed as in other species of *Champsosaurus* [19, 22], with expanded proximal and distal ends. The ulna has a smaller olecranon process than in *Tchoiria klauseni* [50] or *C. gigas* [19], but similar to *C. laramiensis* [9]. Distally, the ulna is expanded.

Portions of the femur, vertebrae, and ribs are also preserved but are eroded (Fig. 10j, m). The vertebrae are generally comparable to other neochoristoderes, showing unfused neurocentral sutures and a prominent ridge along the dorsal midline of the centrum (Fig. 10j, k).

### Inferring choristodere phylogeny

#### Parsimony analysis

**Fig. 11:**
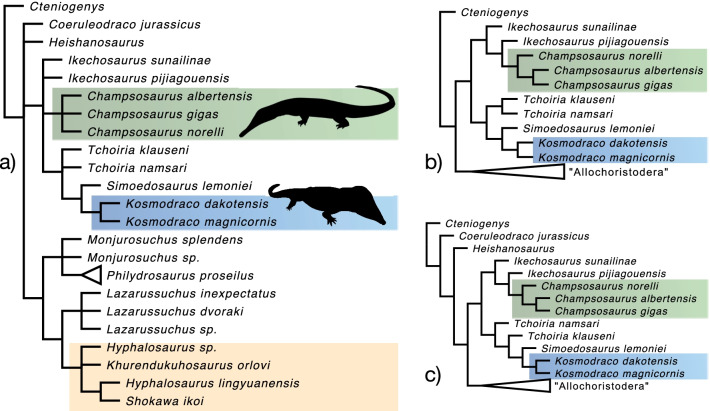
Results of the phylogenetic analysis of choristodere interrelationships under parsimony. **a** Strict consensus topology from the analysis of the phylogenetic matrix under parsimony. Different most parsimonious subtrees **b**, **c** showing conflicting topologies within Neochoristodera, but consistent placement of *Tchoiria* spp. and *Ikechosaurus* spp. as early-diverging simoedosaurids and champsosaurids, respectively. Key clades highlighted (Champsosaurus in green, Kosmodraco in blue, and derived "allochoristoderes" in yellow). Champsosaurus silhouette public domain from phylopic.org

Phylogenetic analysis of a matrix consisting of 32 operational taxonomic units (OTUs) coded for 116 characters produced 4 most parsimonious trees of length 339 (Fig. 11; consistency index = 0.499; retention index = 0.745). A clade containing both species of *Kosmodraco* (64% bootstrap support) and *Simoedosaurus lemoinei* to the exclusion of other neochoristoderes was strongly supported (97% bootstrap support), as was a Neochoristodera including both new taxa (98% bootstrap support). Simoedosauridae, consisting of *Tchoiria*, *Simoedosaurus*, and *Kosmodraco*, had low bootstrap support (9%). *Champsosaurus norelli* was moderately supported as a member of a *Champsosaurus* (46%) and within Champsosauridae (*Champsosaurus* spp. + *Ikechosaurus pijiagouensis* [52], 44% bootstrap support). The relationships of several taxa within Neochoristodera, including both species of *Tchoiria* and *Ikechosaurus sunailinae*, remain unresolved in the strict consensus as in Dong et al. [14]. However, this is clearly due to instability in how these four species are placed within Simoedosauridae and Champsosauridae and not due to disagreements among the most parsimonious trees as to which family of neochoristoderes to which each of these four species pertain (Fig. 11b, c). *Tchoiria* is always resolved as a clade or grade diverging from other simoedosaurids, whereas the *Ikechosaurus* species are found to be a grade leading to the Champsosauridae (Fig. 11b, c). The relationships of members of the “Allochoristodera” [14] also remain incompletely resolved in the consensus, polytomies exist at the bases of the clades containing *Philydrosaurus proseilus* and *Monjurosuchus* spp., all three species of *Lazarussuchus*, and the Hyphalosauridae.

Simoedosauridae was united by 5 characters, and *Kosmodraco* and *Simoedosaurus* were united by eight, the highest number of characters uniting any choristodere clade besides Neochoristodera and *Champsosaurus*. These include several characters relating to the shortening of the rostrum, changes to the palatal dentition, and modifications to the braincase. Within Simoedosauridae, the two species of *Kosmodraco* were united by two characters: [10:1] a maxillary tooth row that terminates at the orbital margin and [29:0] a widened parietal-postinfraorbital zone of contact. Examination of alternate most parsimonious topologies consistently found this sister relationship between *Kosmodraco* and *Simoedosaurus*. However, different trees found *Ikechosaurus sunailinae,* one or both species of *Tchoiria*, or all three of these to be sister to Simoedosauridae. These three species were also supported as outgroups to a clade consisting of Simoedosauridae and the Champsosauridae (synonymous with *Champsosaurus* in my phylogenetic analysis). These results reflect a moderate degree of uncertainty in neochoristodere relationships as inferred by parsimony.

### Bayesian analysis

**Fig. 12:**
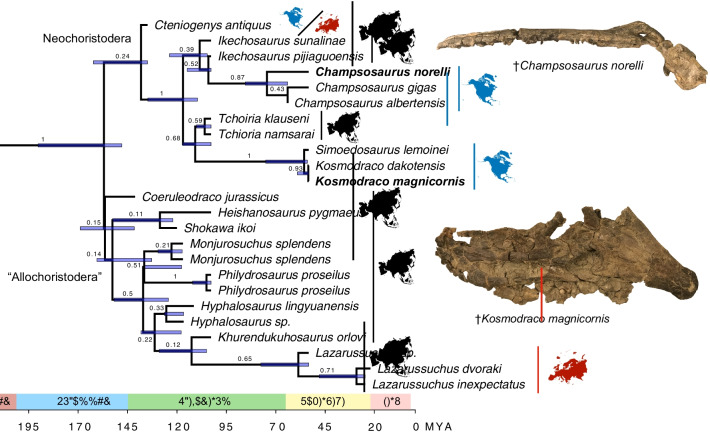
Results of the Bayesian phylogenetic analysis of choristodere interrelationships. Time-calibrated maximum clade credibility tree of choristoderes showing divergence time 95% confidence intervals (blue bars at nodes) and geographic distribution of choristodere species

Analysis of the morphological dataset under a Bayesian framework returned a fully bifurcating combinable components consensus tree (Fig. 12). Tree topology was generally similar to the most parsimonious trees found in the first analysis, with Neochoristodera branching into the longirostrine Champsosauridae and the brevirostrine Simoedosauridae. Whereas the latter clade was strongly supported (posterior value = 1.00). Champsosauridae was only weakly supported with a posterior value of 0.39. This may relate to the wide distribution of longirostry and the associated individual modifications to bones of the cranium associated with this condition among neochoristoderes. Bayesian analysis also returned both species of *Tchioria* as simoeodosaurids, indicating rostral elongation is plesiomorphic for Neochoristodera (Fig. 12). Another major difference between the Bayesian and parsimony results was that, in the former analysis, all non-neochoristodere choristoderes except the Jurassic taxon *Cteniogenys* grouped with “Allochoristodera”, a poorly-known clade only recently recognized by Dong et al. [14]. This clade was given a posterior value of 0.15, indicating a low degree of support. Posterior values for the placement of *Champsosaurus norelli* within *Champsosaurus* and a monophyletic *Kosmodraco* were both high (0.87 and 0.93, respectively).

Time-calibration of the Bayesian maximum credibility tree found Cretaceous divergences among all major clades of neochoristoderes, as well as within several ‘allochoristodere’ lineages (Fig. 12). The split between neochoristoderes and allochoristoderes was estimated to have taken place 175.5 million years ago (Ma; 95% CI: 165.0-213.9 Ma), and the split between Champsosauridae and Simoedosauridae was estimated at 129.4 Ma (95% CI: 120.9-149.8 Ma). Among biogeographic patterns observable across the tree, neochoristoderes show two clear episodes of vicariance among Eurasian and North American lineages that took place during the Late Cretaceous to Paleogene (Fig. 12). One, between the European *Simoedosaurus lemoinei* and the North American *Kosmodraco* spp., was found to take place 58.7 million years ago (95% CI: 56.8-81.1 Ma), whereas the other, between North American *Champsosaurus* spp. and the Chinese species *Ikechosaurus pijagouensis*, was estimated to take place 114.5 million years ago (95% CI: 113-126.9 Ma). Finally, *C. norelli* was estimated to split from other species of *Champsosaurus* 80.4 million years ago (95% CI: 68.3-93.0 Ma).

## Discussion

### Diversity, biogeography, and ecology of the new neochoristoderes

*Kosmodraco magnicornis* and *Champsosaurus norelli* are two of the best-characterized Cenozoic choristoderes and double the diversity and morphological disparity of the clade in the aftermath of the Cretaceous-Paleogene mass extinction. They clarify the evolutionary history of previously described species (i.e., *Kosmodraco dakotensis* gen. et. comb. nov.) and demonstrate that unappreciated choristodere diversity likely lies unrecognized even among previously collected material. Cenozoic choristodere specimens from across the northern hemisphere have been lumped in *Champsosaurus* and *Simoedosaurus* [56], mainly on the basis of general similarities in the postcranial anatomy of these specimens to the two best-known neochoristoderes (e.g., [75]). This study demonstrates that the general dichotomy between longirostrine and brevirostrine choristoderes obscures important anatomical and biogeographic patterns among these groups which arguably warrant generic distinction. For example, the time-calibrated phylogeny of choristodere interrelationships finds that the divergence between *Kosmodraco* and *Simoedosaurus* occurred during the Cretaceous-Paleogene, meaning these clades were isolated on different sides of the Atlantic Ocean for several million years. Along with the number of features distinguishing the American species from the European one, this deep divergence across both space and time warrants generic distinction between these lineages. To this end, I suggest that the various fragmentary postcrania from North America assigned to *Simoedosaurus* [75] be considered Simoedosauridae indeterminate until further osteology study of the postcrania of these large-bodied neochoristoderes is conducted. Such specimens may substantiate further hidden diversity in the choristodere assemblages of the Americas.

Both species represent endemic North American lineages of neochoristoderes, and support the independent dispersal of at least two clades of large-bodied forms into the Americas during the Late Cretaceous-Paleogene (Fig. 12). Biogeographic distinctions between North American and Eurasian Cenozoic choristodere faunas have been tentatively hypothesized based on the presence of different species of *Simoedosaurus* on both continents (e.g., [20, 56]). The new species of simoedosaurid and champsosaurid strongly support the hypothesis that these geographic distinctions are genuine by (1) showing that the extreme rostral modifications in *Kosmodraco dakotensis* is reflective of a larger western North American clade and (2) further expanding the known diversity of the North American *Champsosaurus* diversification event.

Both new species are remarkable for their large body sizes. The skull of *Champsosaurus norelli* approximates the size of YPM VPPU 16240, a large skull of the giant (~ 4 m; [56]) neochoristodere *Champsosaurus gigas* [19]. Ablation of most sutures among braincase and other cranial elements and non-porous external bone texture in the holotype of *C. norelli* strongly implies this individual was at least nearing somatic maturity [60]. Although *Kosmodraco magnicornis* (skull length = 431 mm) is somewhat smaller than *K. dakotensis* (SMM P76.10.1 skull length = 706 mm; [20]), it is still among the largest known choristoderes [56]. Erickson [20] previously suggested the smaller size of the YPM PU cf. *Simoedosaurus* specimens relative to the holotype of *K. dakotensis* implied the former collection consisted of juveniles. However, the developed squamosal and quadratojugal ornamentation, skull roof bone dermal rugosity, and extensive fusion of the skull roof bones in the holotype of *K. magnicornis* strongly suggests this specimen represents a nearly or completely somatically mature individual. A second indicator of similarity in the ontogenetic status of the holotypes of *K. magnicornis* and *K. dakotensis* is that both specimens display similarly sized orbits relative to the rest of the skull. The relative size of the orbit is known to decrease over the course of choristodere ontogeny [82], so an ontogenetic sequence consisting of *K. magnicornis* and *K. dakotensis* would break this established pattern. Thirdly, *K. magnicornis* and *K. dakotensis* show different dental morphologies, contrasting with the ontogenetic stability observed for this feature in other choristoderes [82]. Together with the temporal and geographic separation of *K. magnicornis* (late Tiffanian, Wyoming; [38]) from *K. dakotensis* (middle Tiffanian, North Dakota; [20]), these observations further demonstrate that large choristodere species were a common occurrence throughout Paleogene ecosystems in the northern hemisphere [56].

It is frequently hypothesized that large predatory species can only coexist if they display some degree of niche partitioning, which allows them to sidestep the fitness cost associated with living in the same environment as morphologically similar taxa (e.g., [11, 44, 55]). This ecological contention has been applied to many cases in the fossil record, most famously to large theropod dinosaurs [26, 63]. On its face, the new choristodere fauna appears to support this model of resource partitioning, particularly considered along with the evidence for niche distinctions between coeval crocodylians and choristoderes [56]. The hypothesis that longirostrine and brevirostrine exploited different food sources is primarily based on the distinctions between the resource consumption of extant analogs (i.e., crocodylians, [19, 20, 23, 57, 62]).

I urge a degree of skepticism regarding the application of rigid niche partitioning as an explanation for high predator diversity in Paleocene-Eocene North American ecosystems. This skepticism is based first on the high diversity of similarly-sized aquatic reptiles [56] and large-bodied fishes (i.e., holosteans and acipenseriforms, [3, 40–42]), which contrasts with what would be expected if strict niche partitioning on the basis of size differences was taking place. Second, the possibility remains that phylogenetic history and not the necessity of ecological specialization explains rostral shape disparity in Cenozoic North American choristoderes. These rostral differences could have facilitated coexistence via niche partitioning, but positing competitive exclusion as a causal agent shaping choristodere cranial disparity seems premature. Although the uncertain phylogenetic positions of several taxa (*Ikechosaurus, Tchoiria*) means that quantitative assessment of phylogenetic signal should wait until the relationships of neochoristoderes are better resolved, the presence of longirostrine and brevirostrine clades that existed throughout the northern hemisphere (and only occasionally in sympatry) during Cretaceous-Eocene [19, 20, 56] implicates evolutionary history rather than local ecology as a key driver in the evolution of neochoristodere skull shape.

What can be said is that the extant constituents of these large predatory vertebrate guilds trace the origins of their current diversity to archaic faunas. On a macroevolutionary scale, the diversification of crocodylians, lepisosteids, and other current North American large predatory vertebrates must be understood in the context of distinct, ancient ecological patterns.

### Incomplete morphological convergence in neochoristoderes, alligatoroids, and lepisosteids

**Fig. 13:**
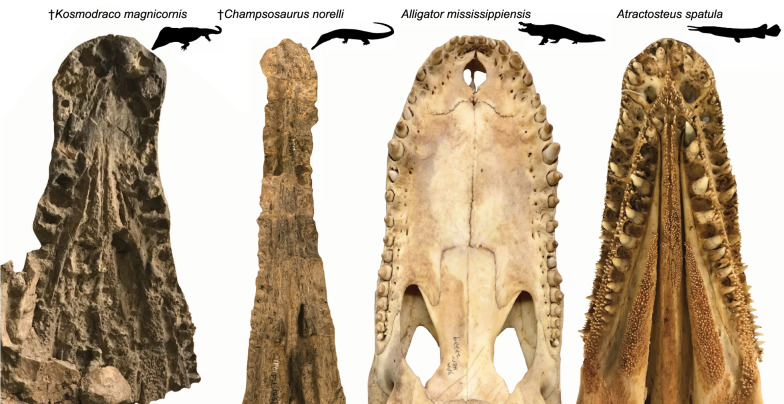
Comparison of the palatal anatomy of choristoderes described in this study with those of extant large-bodied freshwater species found in North America. Note the absence of palatal tooth plates in *Alligator mississippiensis* and the similar placement of the palatal tooth rows in †*K. magnicornis* and *A. spatula*. Also note the similarities in alveolar size changes throughout the tooth rows of *K. magnicornis* and *A. mississippiensis*. ^†^Champsosaurus a n d Alligator silhouettes public domain from phylopic.org.

To the extent that the new choristoderes can be compared with extant species, two lineages stand out in relation to *Kosmodraco*: alligatoroid crocodylians (= alligators and caimans) and lepisosteid holosteans (= gars). Together with choristoderes, these large freshwater predators are consistently found throughout the Late Cretaceous-Paleogene of the northern hemisphere [8, 13, 15, 19, 21, 40, 56, 72]. The morphology of members of these three lineages is broadly comparable: the gar *Atractosteus*, the choristodere *Kosmodraco*, and the alligatoroids (incl. *Alligator, Melanosuchus,* and *Caiman*) all share a short, broad rostrum and a robust skull (Fig. 13).

**Fig. 14:**
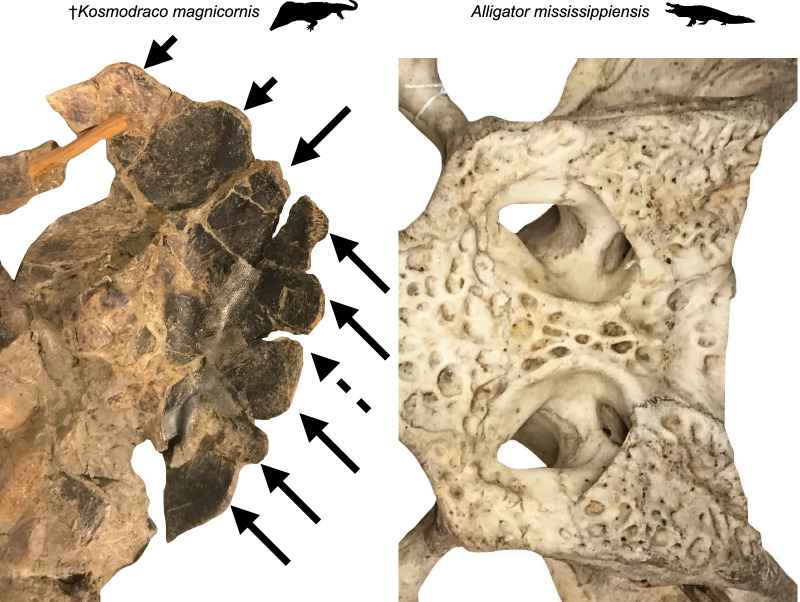
Comparison of posterior skull ornamentation in ^†^*Kosmodraco magnicornis* and *Alligator mississippiensis*. Arrows indicate knob-like ornaments projecting from the squamosal and quadratojugal of ^†^*K. magnicornis*

At the same time, there are notable differences: the three-dimensionally preserved skull of *Kosmodraco magnicornis* is remarkably dorsoventrally shallow relative to the crania of either alligatoroids (i.e., *Alligator*; Fig. 2) or *Atractosteus spatula* [40] and possesses the long postorbital region characteristic of neochoristoderes. Nonetheless, *Kosmodraco magnicornis* shows the raised orbital region found in alligatoroids (Fig. 3), a feature absent in gars [40]. *A. spatula* and *Kosmodraco* share broadened tooth plates on the palate [20], which are absent in crocodylians. Further, the skulls of *A. spatula* and *Kosmodraco* are subtriangular in dorsal view, whereas the skulls of extant North American alligatoroids and their extinct Late Cretaceous-Eocene relatives are subrectangular (e.g., [7, 8, 13]). Finally, the posterior cranial ornamentation present in both species of *Kosmodraco* and accentuated in *K. magnicornis* has no clear analog in either of these other clades (Fig. 14).

As discussed, the postcrania known for *Kosmodraco magnicornis* deviate from the conditions in crocodylians. More than two sacrals are present as in other choristoderes, although they do not overlap as in some champsosaurids and possess distinctive prominences which I interpret as attachment sites for the musculature and ligaments associated with the sacrum and basal caudal region (e.g., [4; 12; 43]). These observations underscore the difficulty of inferring ecological similarities based on morphology alone and show the necessity for biomechanical studies to infer the mechanisms by which these different clades engaged in prey capture (e.g., [58, 62]).

The skull of *Champsosaurus norelli*, like other longirostrine choristoderes, deviates from the robust skulls of alligatoroid and crocodylid crocodylians (Fig. 10) and instead resembles the condition in gavialoids (e.g., [9, 18, 19, 23, 30, 50, 56]). Matsumoto et al. [62] recently documented key differences in the cervical anatomy of longirostrine choristoderes and gavialoids that imply a different set of biomechanical processes took place in the former clade.

### Choristodera as an extinct depauperon

Because of their low taxic diversity across their > 100-million-year evolutionary history (e.g., [23]), choristoderes could be considered an example of a depauperon, a long-lived lineage with a consistently low level of taxonomic diversity. However, it is unclear whether the status of Choristodera as an apparent depauperon reflects a genuine pattern of diversity or represents the results of incomplete sampling of this lineage over certain time bins or excessive lumping of specimens into previously described genera (e.g., [20, 23, 30, 60]). Indeed, the discovery of diverse assemblages of Early Cretaceous choristoderes from Asia challenge the status of Choristodera as a depauperon [14, 23, 24, 56]. Nonetheless, all of these species are members of the ‘Allochoristodera’ [14]. If the Jurassic *Cteniogenys* is also an allochoristodere, Choristodera includes at least two long branches (Fig. 12). One, at the base of Neochoristodera, extends from the Middle Jurassic into the Early Cretaceous, an interval of at least 50 million years. The second leads to all species of the diminutive Cenozoic European choristodere *Lazarussuchus* and is found to track over more than 140 million years of Earth history in the time-calibrated Bayesian maximum clade credibility tree (Fig. 12).

The results of the reexamination of Cenozoic North American neochoristodere faunas presented here contrasts with the expected pattern under the depauperon model. Together, *Kosmodraco* spp., *Champsosaurus* spp., and Paleocene material assigned to *Simoedosaurus* sp. [30] demonstrate that at least seven different neochoristodere species all existed in western North America in the ten million years following the K-Pg mass extinction. They add to a growing body of evidence that Cenozoic neochoristoderes showed a large range of cranial [19, 20, 30, 56, 57] and postcranial [20, 62] morphologies. As such, *Kosmodraco magnicornis* and *Champsosaurus norelli* suggest neochoristoderes represent a largely untapped reservoir of freshwater predator diversity that existed in the aftermath of the bolide impact that ended the Mesozoic. At the same time, the persistence of *Lazarussuchus* into the Neogene tropical environments of Eurasia [56] is very reminiscent of extant small saurian depauperons, such as the tuatara *Sphenodon punctatus* of New Zealand (e.g., [34]) and the limbless dibamids of Mexico, Asia, and Oceania (e.g., [79]).

The case of *Lazarussuchus* underscores the importance of critical reevaluation of the fossil record of choristoderes for illuminating where ‘true’ depauperons lie in the evolutionary tree of this enigmatic clade of reptiles.

## Methods

### Parsimony phylogenetic analysis

In order to test the phylogenetic positions of *Kosmodraco magnicornis* and *Champsosaurus norelli* among choristoderes, I coded the holotypes of both species for the matrix of Matsumoto et al. [60] as modified by Dong et al. [14]. I ran a parsimony analysis in TNT v. 1.5 [39] with characters left unordered. An initial Wagner search over 10 replicates with space for 1000 trees and default parameters for ratchet, tree drift, tree fuse, and sectorial search returned 19 trees of length 353. A subsequent round of traditional bisection-reconnection (TBR) branch swapping with space for 100,000 trees returned a total of 28 most parsimonious trees of length 353. I resampled trees over 100 replicates to calculate bootstrap supports.

### Bayesian phylogenetic analysis

In order to further test the interrelationships of choristoderes under different phylogenetic model frameworks, I conducted a Bayesian analysis of the morphological dataset modified from Dong et al. [14] in the analysis program BEAST 2.5.2 [5]. The fossilized birth–death model was used with a relaxed log-normal clock (1.0 exponential prior for the mean, 0.333 for the standard deviation). The analysis ran over 10 million generations with a 25% burn in. I used Tracer v. 1.7.1 [65] to check for ESS values > 200 and for convergence of posterior and likelihood values. A new dataset of age dates for the taxon sample included in the phylogenetic matrix (Table 1) was used concurrently time-calibrate the maximum clade credibility tree via tip-dating.

**Table 1:** Ages of taxa in phylogenetic analysis

Taxon	Age (Ma)	References
*Youngina*	252.6	[68]
*Prolacerta*	251.902	[78]
*Petrolacosaurus*	298.9	[66]
*Nothosaurus*	233.5	[53]
*Keichousaurus*	240.8	[51]
*Araeoscelis*	272.3	[67] (Early Permian)
*Mesosuchus*	247.2	[10]
*Gephyrosaurus*	190.8	[76]
*Champsosaurus albertensis*	68.3	[16]
*Champsosaurus gigas*	56	[19]
*Champsosaurus norelli*	56	This paper
*Kosmodraco dakotensis*	56	[20]
*Kosmodraco magnicornis*	56	This paper
*Simoedosaurus lemoinei*	56	[74]
*Tchoiria klauseni*	113	[50] (Aptian)
*Ikechosaurus sunailinae*	113	[6] (Aptian)
*Ikechosaurus pijiagouensis*	113	[52]
*Monjurosuchus splendens*	129.4	[59]
*Monjurosuchus splendens*	129.4	[59]
*Hyphalosaurus lingyuanensis*	123	[32]
*Hyphalosaurus* sp.	123	[32]
*Shokawa ikoi*	132.9	[25]
*Cteniogenys antiquus*	150	[80]
*Lazarussuchus inexpectatus*	23.03	[47]
*Lazarussuchus dvoraki*	20	[24]
*Khurendukuhosaurus orlovi*	113	[61]
*Philydrosaurus proseilus*	113	[29]
*Philydrosaurus proseilus*	113	[29]
*Tchoiria namsari*	113	[17]
*Lazarussuchus* sp.	56	[60]
*Coeruleodraco jurassicus*	157.3	[61]
*Heishanosaurus pygmaeus*	113	[14]

## Supplementary Information

**Additional file 1.** Supplementary text and figures.

**Additional file 2.** Supplementary phylogenetic data.

## Data Availability

All data generated for this study is given in the manuscript and additional information. The specimens examined all reside in the vertebrate paleontology and zoology collections of the Yale Peabody Museum of Natural History, a public repository in New Haven, CT.

## Abbreviations

SMM: Science Museum of Minnesota, Saint Paul, MN, USA
YPM VPPU: Princeton University Vertebrate Paleontology Collections in the Yale Peabody Museum of Natural History, New Haven, CT, USA
