# Correction: High morphological disparity in a bizarre Paleocene fauna of predatory freshwater reptiles

**DOI:** 10.1186/s12862-022-02000-1

**Published:** 2022-04-15

**Authors:** Chase Doran Brownstein

**Affiliations:** 1 Stamford Museum and Nature Center, Stamford, CT USA; 2 Department of Ecology and Evolutionary Biology, Yale University, New Haven, CT USA

## Correction to: BMC Ecology and Evolution (2022) 22:34 https://doi.org/10.1186/s12862-022-01985-z

Following the publication of the original article [1], we were notified that due to an upload issue, Figure 12 looked distorted. This has now been replaced with a clearer version.

The original article has been corrected.
