# Supplementary material for: High morphological disparity in a bizarre Paleocene fauna of predatory freshwater reptiles
Source: BMC Ecol Evol. 2022 Mar 21;22:34. doi: 10.1186/s12862-022-01985-z (PMC8935759; doi:10.1186/s12862-022-01985-z)
Supplement: Supplementary file 1 — Additional file 1. Supplementary text and figures. [file 12862_2022_1985_MOESM1_ESM.pdf]

Supplement to: High morphological disparity in a bizarre Paleocene fauna of predatory freshwater reptiles

Chase Doran Brownstein[1, 2]*
[1]Stamford Museum and Nature Center, Stamford CT, USA
[2]Department of Ecology and Evolutionary Biology, Yale University, New Haven CT, USA
*Corresponding author, **chase.brownstein@yale.edu**

I. Measurements.

Kosmodraco magnicornis measurements.
maximum anteroposterior skull length: 431 mm
minimum mediolateral width of rostrum: 47 mm
maximum anteroposterior scapula length: 115 mm
Champsosaurus norelli measurements.
maximum anteroposterior skull length:
minimum mediolateral width of rostrum:
maximum proximodistal length of humerus:
maximum proximodistal length of ulna:

II. Apomorphies found in parsimony analysis of choristoderes.

Apomorphies optimized for individual clades in the parsimony analysis (not converted from TNT format, so char 0 below = char 1 in matrix). Key groups highlighted in bold.

Youngina :
  No autapomorphies
Prolacerta :
  Char. 3: 0 --> 1
  Char. 53: 0 --> 1
  Char. 67: 0 --> 1
  Char. 76: 0 --> 1
  Char. 80: 0 --> 2
  Char. 90: 0 --> 1
  Char. 94: 0 --> 1
  Char. 108: 0 --> 1
  Char. 111: 0 --> 1
  Char. 112: 0 --> 1
  Char. 114: 0 --> 2
Petrolacosaurus :
  Char. 40: 1 --> 0
  Char. 45: 1 --> 0
  Char. 54: 1 --> 0
Nothosaurus :
  Char. 9: 0 --> 2

Char. 25: 0 --> 2
Char. 30: 0 --> 2
Char. 61: 0 --> 1
Char. 81: 0 --> 1
Char. 102: 2 --> 1
Char. 105: 1 --> 0
Char. 108: 1 --> 0
Keichousaurus :
Char. 16: 0 --> 2
Char. 17: 1 --> 0
Char. 28: 0 --> 1
Char. 34: 1 --> 0
Char. 36: 1 --> 0
Char. 65: 1 --> 2
Char. 80: 0 --> 2
Araeoscelis :
Char. 6: 0 --> 12
Char. 32: 0 --> 2
Char. 33: 1 --> 0
Char. 41: 1 --> 0
Char. 43: 0 --> 1
Char. 74: 0 --> 1
Char. 80: 0 --> 2
Char. 81: 0 --> 1
Mesosuchus :
Char. 0: 0 --> 1
Char. 3: 0 --> 3
Char. 11: 2 --> 1
Char. 16: 0 --> 1
Char. 74: 0 --> 1
Char. 80: 0 --> 1
Char. 100: 0 --> 2
Gephyrosaurus :
Char. 28: 0 --> 1
Char. 45: 1 --> 0
Char. 71: 0 --> 3
Char. 73: 0 --> 1
Champs_albertensis :
Char. 75: 1 --> 3
Champsosaurus_gigas :
Char. 62: 1 --> 3
Champsosaurus_norelli :
Char. 56: 1 --> 2

Kosmodraco_dakotensis :
No autapomorphies
Kosmodraco_magnicornis :
No autapomorphies
Simoedosaurus_lemoniei :
Char. 53: 1 --> 2
Tch__IGM1_8 :
Char. 13: 0 --> 3
Char. 26: 1 --> 0
Ik__IVPP9611_3 :
Char. 40: 1 --> 0
Char. 44: 0 --> 1
Char. 45: 1 --> 2
Char. 60: 12 --> 0
Char. 75: 1 --> 2
Ik__IVPP13283 :
Char. 28: 1 --> 0
Char. 86: 1 --> 0
M__DR003 :
Char. 28: 0 --> 1
M__BMNHC073 :
Char. 24: 0 --> 1
Char. 98: 1 --> 0
H__IVPP_11075 :
Char. 96: 0 --> 1
Char. 107: 0 --> 1
H__IVPP_14560 :
Char. 83: 0 --> 1
Shokawa :
Char. 104: 1 --> 0
Char. 110: 0 --> 1
Char. 111: 1 --> 0
Cteniogenys :
Char. 0: 0 --> 1
Char. 1: 0 --> 1
Char. 9: 0 --> 1
Char. 16: 0 --> 2
L_inexpectatus :
Char. 85: 0 --> 1
Char. 93: 1 --> 0
L_dvoraki :
Char. 68: 0 --> 1
Khurendukuhosaurus :

Char. 71: 0 --> 1
Char. 84: 0 --> 1
Char. 100: 1 --> 2
Ph__PKUP_2001 :
No autapomorphies
Ph__LMPC021 :
No autapomorphies
Tch__PIN3386_1 :
Char. 108: 1 --> 0
Lazarussuchus_sp. :
Char. 105: 1 --> 0
Coeruleodraco_jurassicus :
Char. 13: 0 --> 2
Char. 17: 1 --> 2
Char. 65: 1 --> 2
Char. 77: 1 --> 0
Char. 108: 1 --> 0
Char. 114: 0 --> 2
Heishanosaurus :
Char. 31: 0 --> 1
Char. 77: 1 --> 0
Char. 82: 1 --> 0
Char. 95: 1 --> 0
Node 33 :
No synapomorphies
Node 34 :
Char. 10: 1 --> 0
Char. 17: 1 --> 0
Node 35 :
Char. 6: 3 --> 0
Char. 26: 1 --> 0
Node 36 :
Char. 2: 0 --> 1
Char. 10: 1 --> 2
Char. 11: 2 --> 3
Char. 15: 0 --> 1
Char. 43: 0 --> 1
Char. 45: 1 --> 3
Char. 52: 0 --> 1
Char. 78: 0 --> 1
Char. 107: 0 --> 2
Char. 109: 0 --> 1
Node 37 :

Char. 8: 0 --> 1
Char. 26: 0 --> 1
Char. 46: 0 --> 1
Char. 47: 0 --> 1
Char. 48: 0 --> 1
Char. 49: 0 --> 1
Char. 77: 0 --> 1
Char. 92: 0 --> 1
Char. 93: 0 --> 1
Char. 99: 0 --> 1
Char. 102: 0 --> 2
Node 38 :
Char. 34: 0 --> 1
Char. 36: 0 --> 1
Char. 76: 0 --> 1
Char. 94: 0 --> 1
Char. 101: 0 --> 1
Char. 106: 0 --> 1
Char. 108: 0 --> 1
Node 39 :
Char. 11: 0 --> 2
Char. 27: 0 --> 3
Char. 44: 0 --> 2
Char. 104: 0 --> 1
Char. 105: 0 --> 1
Node 40 (*Champsosaurus*):
Char. 9: 2 --> 1
Char. 13: 0 --> 1
Char. 19: 0 --> 1
Char. 35: 0 --> 2
Char. 37: 1 --> 2
Char. 51: 1 --> 2
Char. 52: 0 --> 1
Char. 57: 0 --> 2
Char. 69: 0 --> 1
Char. 70: 0 --> 1
Char. 80: 0 --> 1
Char. 82: 1 --> 2
Node 41 :
Char. 0: 0 --> 1
Char. 1: 0 --> 1
Char. 3: 0 --> 1
Char. 7: 0 --> 2

Char. 9: 0 --> 2
Char. 12: 0 --> 1
Char. 14: 0 --> 1
Char. 16: 0 --> 1
Char. 18: 1 --> 2
Char. 20: 2 --> 1
Char. 25: 0 --> 2
Char. 28: 0 --> 1
Char. 29: 0 --> 12
Char. 31: 0 --> 1
Char. 56: 0 --> 1
Char. 58: 0 --> 1
Char. 62: 2 --> 1
Char. 72: 0 --> 1
Char. 73: 0 --> 1
Char. 74: 0 --> 1
Char. 79: 0 --> 1
Char. 83: 0 --> 1
Char. 87: 0 --> 1
Char. 88: 0 --> 1
Char. 89: 0 --> 1
Char. 96: 0 --> 1
Char. 109: 0 --> 1
Char. 112: 0 --> 1

Node 42 :
Char. 22: 0 --> 1
Char. 59: 0 --> 1
Char. 61: 0 --> 1
Char. 67: 0 --> 1
Char. 86: 0 --> 1
Char. 111: 0 --> 1

Node 43 :
Char. 5: 0 --> 1
Char. 20: 0 --> 2
Char. 23: 0 --> 1
Char. 30: 0 --> 1
Char. 37: 0 --> 1
Char. 39: 0 --> 1
Char. 44: 2 --> 0
Char. 55: 0 --> 1
Char. 70: 1 --> 0

Node 44 (*Kosmodraco*):
Char. 9: 2 --> 1

Char. 28: 1 --> 0
Node 45 (*Simoedosaurus+Kosmodraco*):
Char. 11: 2 --> 1
Char. 12: 1 --> 0
Char. 30: 1 --> 2
Char. 44: 0 --> 1
Char. 45: 1 --> 2
Char. 51: 1 --> 2
Char. 58: 1 --> 2
Char. 65: 1 --> 2
Node 46 (Simoedosauridae) :
Char. 7: 2 --> 1
Char. 35: 0 --> 1
Char. 71: 0 --> 1
Char. 74: 1 --> 0
Char. 84: 0 --> 1
Node 47 :
Char. 11: 2 --> 1
Char. 84: 0 --> 1
Char. 108: 1 --> 0
Char. 114: 0 --> 2
Node 48 :
Char. 29: 0 --> 2
Char. 30: 1 --> 2
Char. 32: 01 --> 2
Char. 33: 2 --> 0
Char. 69: 0 --> 1
Char. 110: 1 --> 0
Node 49 :
Char. 103: 0 --> 1
Char. 105: 1 --> 0
Node 50 :
Char. 78: 0 --> 1
Char. 82: 1 --> 0
Char. 100: 0 --> 1
Char. 109: 0 --> 1
Node 51 :
Char. 15: 0 --> 1
Char. 17: 1 --> 2
Char. 18: 1 --> 2
Char. 115: 1 --> 0
Node 52 :
Char. 20: 2 --> 1

Char. 76: 1 --> 0
Char. 77: 1 --> 0
Char. 90: 2 --> 0
Char. 92: 1 --> 2
Char. 111: 1 --> 0
Node 53 :
Char. 6: 0 --> 2
Char. 21: 0 --> 1
Char. 22: 1 --> 0
Char. 25: 0 --> 1
Char. 27: 2 --> 1

III. Supplementary Figures.

Figure S1. Additional views and interpretations of the cranial anatomy of *Kosmodraco magnicornis*. Skull (a, c) and interpretive drawing of skull (b, d) in dorsal (a, b) and ventral (c, d) views.

Figure S2. Comparisons between *Champsosaurus norelli* and YPM VPPU 16240, a large specimen of *Champsosaurus gigas*. Skull of *Champsosaurus norelli* in (a) dorsal and (c) ventral views. Skull YPM VPPU 16240 in (b) dorsal and (d) ventral views.

Figure S3. Time-calibrated Bayesian topology. Numbers indicate posterior values for particular nodes.

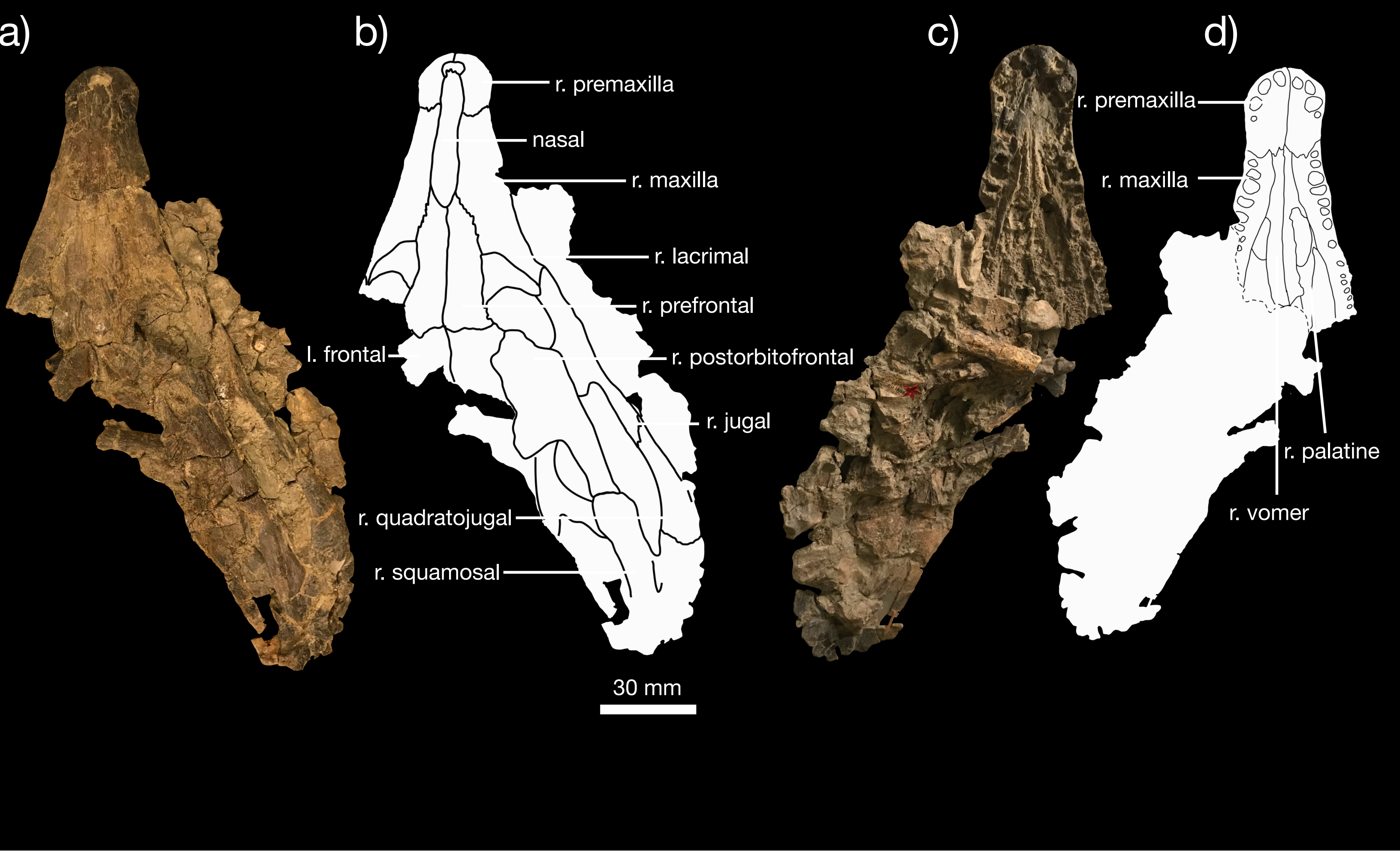
a)
b)
c)
d)
r. premaxilla
nasal
r. maxilla
r. lacrimal
r. prefrontal
l. frontal
r. postorbitofrontal
r. jugal
r. quadratojugal
r. squamosal
30 mm
r. premaxilla
r. maxilla
r. palatine
r. vomer

Figure S1.

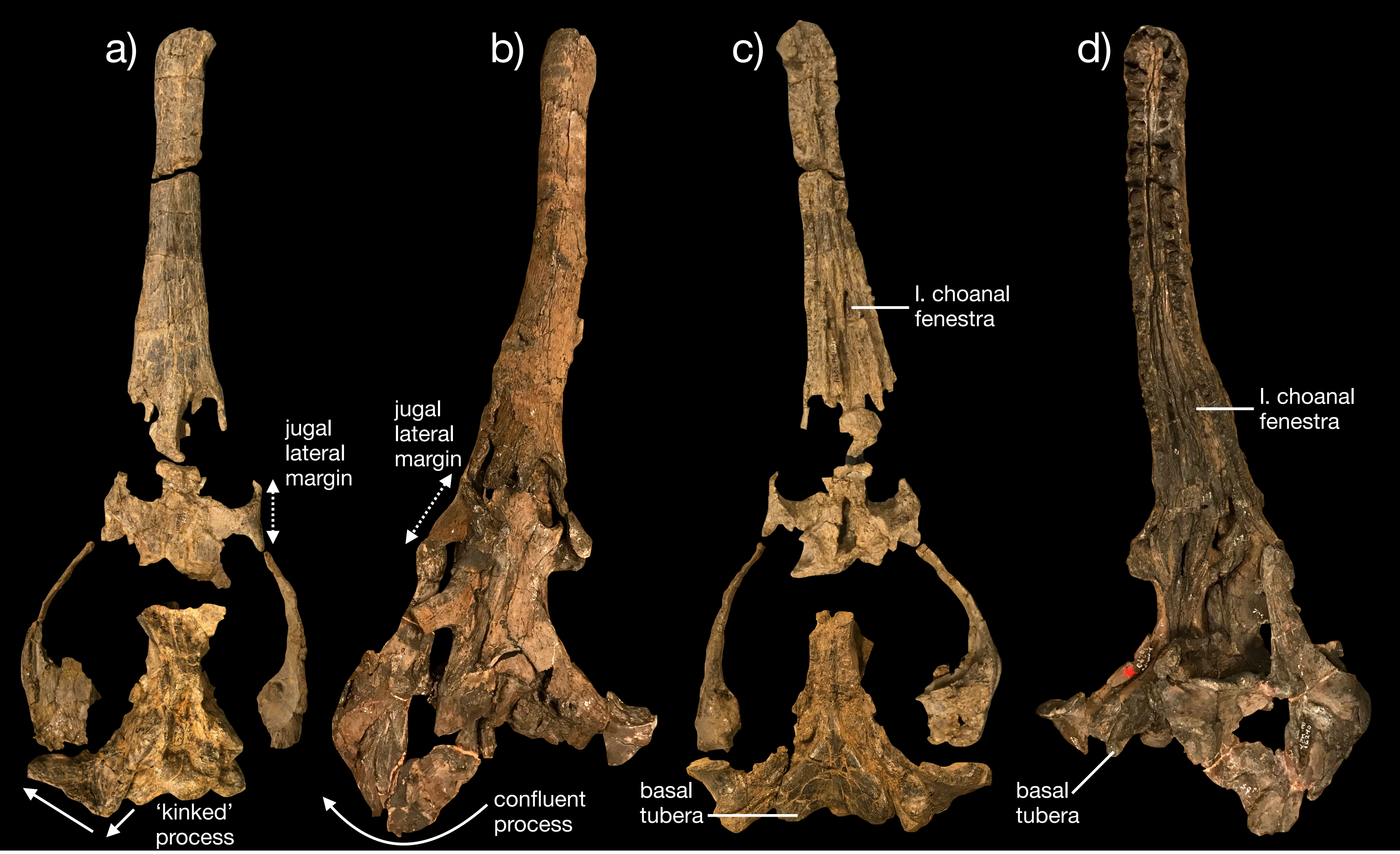

a)
b)
c)
d)
jugal
lateral
margin
jugal
lateral
margin
l. choanal
fenestra
l. choanal
fenestra
'kinked'
process
confluent
process
basal
tubera
basal
tubera

Figure S2.

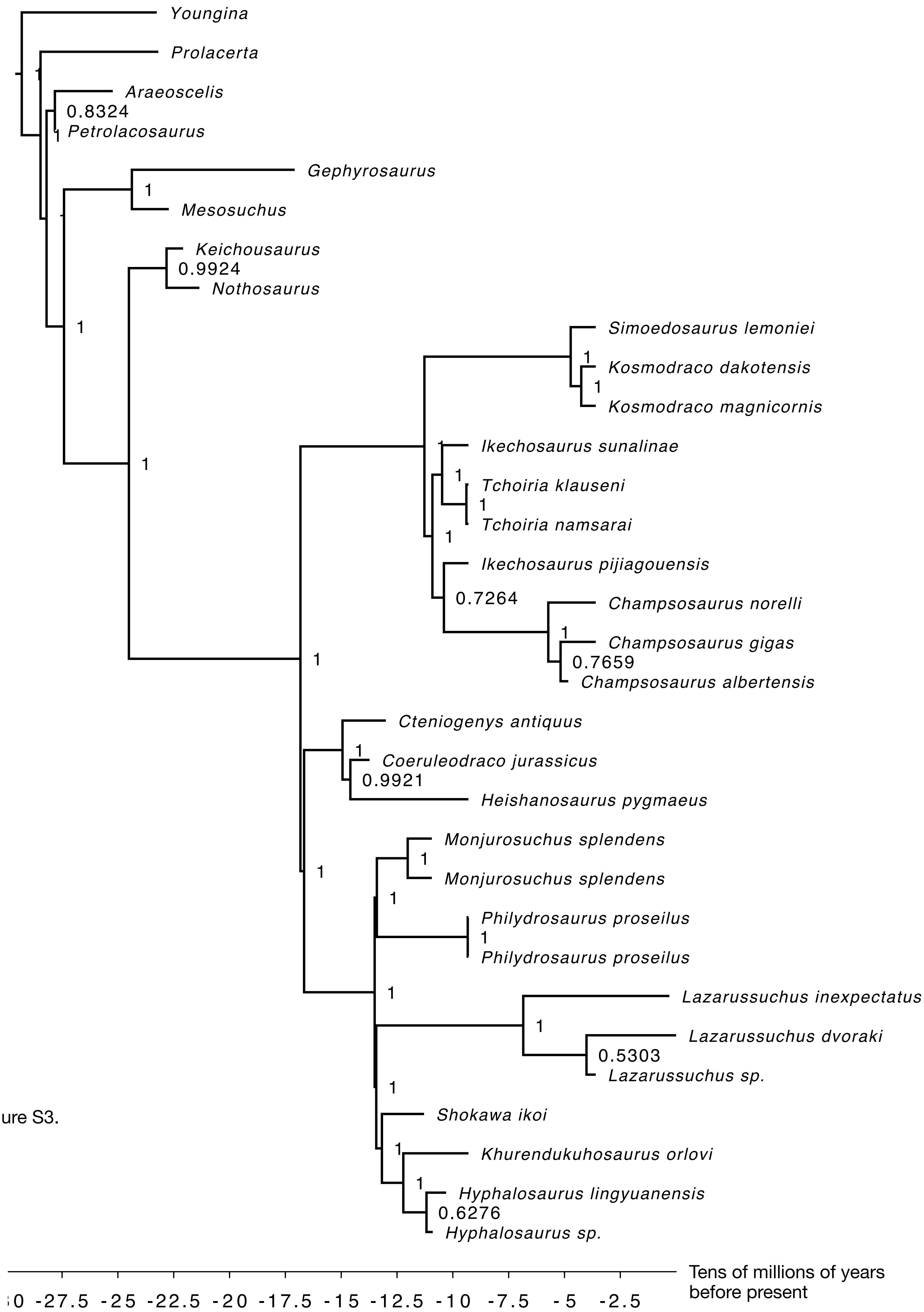

Youngina
Prolacerta
1
Araeoscelis
0.8324
Petrolacosaurus
1
Gephyrosaurus
1
Mesosuchus
Keichousaurus
0.9924
Nothosaurus
1
Simoedosaurus lemoniei
1
Kosmodraco dakotensis
1
Kosmodraco magnicornis
1
Ikechosaurus sunalinae
1
Tchoiria klauseni
1
Tchoiria namsarai
1
Ikechosaurus pijiagouensis
0.7264
Champsosaurus norelli
1
Champsosaurus gigas
0.7659
Champsosaurus albertensis
1
Cteniogenys antiquus
1
Coeruleodraco jurassicus
0.9921
Heishanosaurus pygmaeus
Monjurosuchus splendens
1
Monjurosuchus splendens
1
1
Philydrosaurus proseilus
1
Philydrosaurus proseilus
1
Lazarussuchus inexpectatus
1
Lazarussuchus dvoraki
0.5303
Lazarussuchus sp.
1
Shokawa ikoi
1
Khurendukuhosaurus orlovi
1
Hyphalosaurus lingyuanensis
0.6276
Hyphalosaurus sp.
-27.5 -25 -22.5 -20 -17.5 -15 -12.5 -10 -7.5 -5 -2.5
Tens of millions of years before present

Figure S3.
